# Performance Metrics of Substance Use Disorder Care Among Medicaid Enrollees in New York, New York

**DOI:** 10.1001/jamahealthforum.2022.1771

**Published:** 2022-07-01

**Authors:** Margarita Alegría, Irene Falgas-Bague, Marie Fukuda, Jenny Zhen-Duan, Cole Weaver, Isabel O’Malley, Timothy Layton, Jacob Wallace, Lulu Zhang, Sheri Markle, Charles Neighbors, Pat Lincourt, Shazia Hussain, Marc Manseau, Bradley D. Stein, Nancy Rigotti, Sarah Wakeman, Martha Kane, A. Eden Evins, Thomas McGuire

**Affiliations:** 1 Disparities Research Unit, Massachusetts General Hospital, Boston; 2 Department of Medicine, Harvard Medical School, Boston, Massachusetts; 3 Department of Psychiatry, Harvard Medical School, Boston, Massachusetts; 4 Department of Health Care Policy, Harvard Medical School, Boston, Massachusetts; 5 Yale School of Public Health, New Haven, Connecticut; 6 Grossman School of Medicine, New York University, New York; 7 Wagner School of Public Service, New York University, New York; 8 New York State Office of Alcoholism and Substance Abuse Services, Albany, New York; 9 New York State Office of Mental Health, New York; 10 RAND Corporation, Pittsburgh, Pennsylvania; 11 Division of General Internal Medicine, Massachusetts General Hospital, Boston; 12 Tobacco Research and Treatment Center, Massachusetts General Hospital, Boston; 13 Substance Use Disorder Initiative, Massachusetts General Hospital, Boston; 14 Addictions Services Unit, Massachusetts General Hospital, Boston; 15 Center for Addiction Medicine, Massachusetts General Hospital, Boston

## Abstract

**Importance:**

There is limited evaluation of the performance of Medicaid managed care (MMC) private plans in covering substance use disorder (SUD) treatment.

**Objective:**

To compare the performance of MMC plans across 19 indicators of access, quality, and outcomes of SUD treatment.

**Design, Setting, and Participants:**

This cross-sectional study used administrative claims and mandatory assignment to plans of up to 159 016 adult Medicaid recipients residing in 1 of the 5 counties (boroughs) of New York, New York, from January 2009 to December 2017 to identify differences in SUD treatment access, patterns, and outcomes among different types of MMC plans. Data from the latest years were received from the New York State Department of Health in October 2019, and analysis began soon thereafter. Approximately 17% did not make an active choice of plan, and a subset of these (approximately 4%) can be regarded as randomly assigned.

**Exposures:**

Plan assignment.

**Main Outcomes and Measures:**

Percentage of the enrollees achieving performance measures across 19 indicators of access, process, and outcomes of SUD treatment.

**Results:**

Medicaid claims data from 159 016 adults (mean [SD] age, 35.9 [12.7] years; 74 261 women [46.7%]; 8746 [5.5%] Asian, 73 783 [46.4%] Black, and 40 549 [25.5%] White individuals) who were auto assigned to an MMC plan were analyzed. Consistent with national patterns, all plans achieved less than 50% (range, 0%-62.1%) on most performance measures. Across all plans, there were low levels of treatment engagement for alcohol (range, 0%-0.4%) and tobacco treatment (range, 0.8%-7.2%), except for engagement for opioid disorder treatment (range, 41.5%-61.4%). For access measures, 4 of the 9 plans performed significantly higher than the mean on recognition of an SUD diagnosis, any service use for the first time, and tobacco use screening. Of the process measures, total monthly expenditures on SUD treatment was the only measure for which plans differed significantly from the mean. Outcome measures differed little across plans.

**Conclusions and Relevance:**

The results of this cross-sectional study suggest the need for progress in engaging patients in SUD treatment and improvement in the low performance of SUD care and limited variation in MMC plans in New York, New York. Improvement in the overall performance of SUD treatment in Medicaid potentially depends on general program improvements, not moving recipients among plans.

## Key Points

**Question:**

How do Medicaid managed care (MMC) plans compare in terms of covering substance use disorder (SUD) treatment across indicators of access, quality, and outcomes in New York, New York?

**Findings:**

In this cross-sectional study of administrative records from 159 016 adult Medicaid recipients who did not choose a plan and who could be regarded as randomly assigned across plans, engagement in SUD treatment was low across all plans. There was significant expenditure variation among plans, with several plans with low spending on SUD services, and outcomes differed little across plans, with low performance.

**Meaning:**

The study findings suggest a need for improved monitoring of the performance of Medicaid private plans.

## Teaser

This cross-sectional study examines the performance of Medicaid managed care plans across 19 indicators of access, quality, and outcomes of substance use disorder treatment.

## Introduction

Lack of substance use disorder (SUD) treatment contributes to adverse personal and social outcomes,^1^ with an estimated 100 431 drug overdose deaths in the US from April 2020 to 2021.^2^ Yet, of the nearly 20 million US adults meeting SUD diagnostic criteria, only 1 in 10 receives specialty treatment.^3^ People with low incomes with SUD are among the most vulnerable, with elevated mortality risk and higher death rates of COVID-19.^4^ Access to high-quality addiction care is especially limited.^5,6^ Medicaid policies can dictate financial access to services^7^; however, nonfinancial barriers are common^8^ and include clinician shortages^9^ and low reimbursements.^10,11^ Quality metrics measure the performance of plans against payer and beneficiary expectations of quality and outcomes and can identify whether plans deliver on coverage standards.

After the implementation of the Affordable Care Act, increased Medicaid enrollment did not translate into increased SUD treatment.^12^ Medicaid in most states has inadequate coverage to effectively treat SUD (eg, medication for opioid use disorders^13^), and services may be more limited within Medicaid managed care (MMC) plans with restrictive coverage options.^13^ There are substantial variabilities in plan offerings (eg, residential treatments, medication) that restrict access to the full care continuum.^14^ Nonetheless, some argue that SUD treatment and outcomes in MMC will be better than fee for service given the potential for better coordination and integration.^15^

New York State (NYS) operates one of the country’s largest Medicaid programs,^16^ which has privatized health insurance and management.^17^ More than three-quarters of Medicaid beneficiaries are enrolled in MMC plans,^16^ with much unknown about the associations of different approaches with access, quality, and outcomes.^18,19,20^ It is difficult to interpret observed differences in state metrics, partially because of differences in the enrollee health status of the populations in different plans.^21^

To overcome this hurdle, we leveraged a natural experiment, the essentially random assignment of a subset of Medicaid recipients who do not actively choose a plan. We used administrative Medicaid data from January 2009 to December 2017 from New York, New York, where two-thirds of the Medicaid population of NYS resides, to identify how MMC plans compare in SUD treatment coverage across access and quality indicators. The null hypothesis was that there would be no performance difference across plans because of similar funding and operations.^22^

Managed care involves utilization management and clinician contracting. Investment in clinician recruitment and retention varies by plan and is associated with clinician type, specialty facility range, access to in-network clinicians, and clinician credentials. In a 2017 Kaiser survey,^23^ 70% of MMC plans with in-network psychiatric nurse practitioners reported difficulty recruiting, and only two-thirds with outpatient services contracted clinics that dispensed opioid use disorder medications. Most MMC plans are private nonprofit plans, making performance crucial to ensure the best use of scarce public funds for SUD services and outcomes. The main objective of this study was to compare MMC plans performance across 19 indicators of SUD treatment access, quality, and outcomes.

## Methods

### Study Design and Data

The study followed the Strengthening the Reporting of Observational Studies in Epidemiology (STROBE) reporting guideline for cross-sectional study reporting (eTable 5 in the Supplement). Data from the latest years were received from NYS Department of Health in October 2019. Analysis began soon afterwards. The institutional review board of the Harvard Faculty of Medicine determined that this secondary analysis was not considered human participants research; thus, informed consent was waived. The Mass General Brigham institutional review board approved the collaboration with Harvard Medical School as part of a protocol, including additional qualitative analysis not described in this article.

Since October 2011, newly enrolled New York Medicaid recipients have 30 days to choose a plan. According to the study data, 17% of beneficiaries fail to make an active choice of plan and are auto assigned. A subset can be regarded as being randomly assigned across eligible plans with equal probability. Not all of the 17% of beneficiaries fit this profile because of previous enrollment/family enrollment. The autoassigned subset has the advantage of supporting more definitive statements about plan performance difference but the disadvantage of possibly being unrepresentative of the broader enrollee population.

We restricted the sample to adults aged 18 to 64 years with at least 6 months of plan enrollment postassignment who were living in 1 of the 5 boroughs of New York, New York. We began with a sample of 246 230 individuals who were auto assigned during the 2009 to 2017 period and determined that 159 016 (64.6%) could be regarded as assigned randomly. A subset qualified for some indicators, such as rapid readmission.

We studied the first 6 months of enrollment for each autoassigned beneficiary. A plan was eligible for random assignment if it met state-defined quality standards,^24^ prevention quality indicators^25^ and regulatory compliance measures. We studied 9 plans that were eligible for autoassignment.

Administrative records from the NYS Department of Health included plan enrollment information and insurance claims from 2009 to 2017. For each recipient, we observed limited demographic information, including race and ethnicity, monthly enrollment, and Medicaid-covered claims. Enrollment data included an indicator for autoassignment. Health services claims included detailed patient diagnoses, procedures, clinician identifiers, and insurer payment. The NYS Department of Health standardizes coding of diagnosis and procedures based on Berenson-Eggers Type of Service codes^26^ and Healthcare Common Procedure Coding System for outpatient data. For inpatient data, we used the Clinical Classifications Software (Healthcare Cost and Utilization Project)^27^ to assign each inpatient admission based on a primary diagnosis. Medications were classified according to the National Drug Code. Expenditure data were based on pay fields.

### Performance Indicators

Performance measures were intended to help states establish accountability, promote quality improvement efforts, increase awareness of priority monitoring areas, and inform managed care value-based payment arrangements.^28^ We selected 19 indicators^29,30,31^ based on a literature review that were measurable and informative about SUD treatment (eTable 1 in the Supplement). We refined the selection and definition with clinicians and health administrators with SUD treatment expertise, including pharmacology and claims billing. Clinical experts reviewed codes and grouping used in identifying services, treatments, medications, and diagnosis. The conceptual framework of Donabedian^32^ guided the selection of indicators,^33^ focusing on structure (ie, access), process (ie, patterns of care), and outcomes. Table 1 contains indicator definitions. eTable 1 in the Supplement contains full algorithm construction information. The SUD sample was identified by prescription (with National Drug Code codes), diagnostic code (*International Classification of Diseases, Ninth Revision, Clinical Modification* [*ICD-9*] and *ICD-10* codes), or procedure. The sample size varied by performance indicator depending on selection criteria. eTable 2 in the Supplement contains detailed definitions of SUD sample identification by indicator.

**Table 1.** Indicator Definitions

Indicator	Denominator	Numerator
Access		
Screening or first assessment visits: outpatient care	Had an outpatient claim	First outpatient claim included either an SUD screening or SUD assessment claims
Presence of an SUD diagnosis in claim	Everyone in the sample	Had at least 1 SUD diagnosis in claim
Any SUD service use for the first time	Everyone in the sample	Had at least 1 SUD service claim and continuously enrolled in Medicaid for 2 mo before and 1 mo after their first SUD service claim
Tobacco screening	Everyone in the sample	Received a tobacco screen
Patterns of care		
Follow-up after withdrawal management (14 d)	Had a withdrawal management episode (withdrawal treatments within 3 d were grouped together in the sample episode)	Follow-up treatment claim, SUD service claim, or prescription must have taken place between 1 and 14 d after discharge, have an SUD diagnosis, and not represent a new withdrawal management episode
SUD Tx		
Initiation	No. of individuals with index visit associated with an alcohol/opioid dependence/abuse diagnosis after 60-d clean period with no SUD claims (negative diagnosis history)	Initiated SUD treatment within 14 d of diagnosis
Engagement	Had SUD code combinations (eg, an outpatient visit with a diagnosis of alcohol abuse) and a negative diagnosis history (60 d)	Initiated SUD treatment within 14 d of diagnosis and had 2 or more SUD code combinations within 29 d of their first postassignment SUD claim. If the initiation of SUD treatment was not a MAT dispensing event, the recipient had 1 or more MAT-related claims within 33 d after the initiation event
Engagement		
Alcohol pharmacotherapy	Had index visit (first SUD claim occurs) that was associated with an alcohol dependence/abuse diagnosis and occurred after a 60-d clean period with no SUD claims	Initiated pharmacotherapy with at least 1 prescription for alcohol treatment medication within 30 d following the type of the index visit
Opioid pharmacotherapy	Had index visit (first SUD claim occurs) that was associated with an opioid dependence/abuse diagnosis and occurred after a 60-d clean period with no SUD claims	Initiated pharmacotherapy with at least 1 prescription for opioid treatment medication within 30 d following the type of the initial diagnostic visit
Smoking cessation engagement	Identified as smokers by national drug codes	Initiated smoking cessation services
SUD Tx engagement: follow-up within 30 d after ED visit	Had an ED visit with a principal diagnosis of AOD abuse or dependence. Excluded ED visits followed by an admission to an acute or nonacute inpatient care setting on the date of the ED visit or within the 30 d after the ED visit regardless of principal diagnosis for the admission	Had an eligible follow-up visit
Identification of comorbidity with mental illness	Had at least 1 SUD claim (treatment, service, or diagnosis)	Had a diagnosis of mental health condition
Monthly expenditures on SUD Tx (mean, including zeros)	Mean of the members' average monthly expenditure on SUD	
% Of nonzero expenditures on SUD Tx	% Of members with nonzero expenditure on SUD	
Receipt of psychosocial interventions	Had at least 1 SUD claim (treatment, service, or diagnosis)	Received at least 1 psychosocial intervention
Outcomes		
Rapid readmission to inpatient SUD care 2-30 d postdischarge	Had an acute SUD admission	Had a readmission to the same level of care within 2-30 d
Relapse (2-30 d)	Received SUD treatment	Had at least 1 relapse event
Treatment continuation	Received SUD treatment	Continued treatment for 3 mo or more
Social connectedness (peer services)	Had at least 1 SUD claim (treatment, service, or diagnosis)	Received any peer-support services

Abbreviations: AOD, alcohol and other drug; ED, emergency department; MAT, medication-assisted treatment; SUD, substance use disorder; Tx, treatment.

### Statistical Analysis

We tested for mean differences with a set of linear regressions, 1 for each performance indicator, including dummy variables for each of the 9 plans. Because a few plans did not qualify for random assignment owing to regulatory policies, we regarded randomization as occurring within a county in a given month. Each regression included county-year-month fixed effects and standard errors clustered at this level. Although for each person the indicator by outcome assumed a value of 0 or 1, we demeaned the data by subtracting the overall sample mean at the individual level before running regressions. After demeaning, we dropped a constant term from each regression and used a plan dummy variable to capture plan effects. The statistical significance of plan differences was then tested against the overall mean with a *t* test. For each indicator, we tested whether plans as a group were associated with performance with an *F* test. Because we grouped data into 3 periods, we subtracted the sample mean for each period separately. Demeaning by period adjusted for overall trends in performance, allowing us to isolate plan differences. Nevertheless, as a robustness check, we ran regressions with and without an indicator for each period. The magnitude and significance of the period indicators were very small and had virtually no association with the coefficients of interest (unpublished data; Alegria et al, 2022). Finally, we topcoded spending at the 99th percentile for each period, a common accommodation to skewness in modeling expenditures. Statistical analysis was conducted using Stata, version 15.1 (StataCorp), and statistical significance was set at α = .05.

## Results

Table 2^34^ compares the autoassigned sample with the entire state to assess the generalizability of the results. The mean (SD) age was 35.9 (12.7) years, and there were 74 204 female participants (46.7%). The next set of variables reported information on race and ethnicity, with the New York, New York sample containing fewer White individuals (40 600 [25.5%]). Before random assignment, each autoassigned recipient would generally have spent 30 to 60 days in New York’s fee-for-service Medicaid plan. We used this data to determine that 11.0% of the sample had some indication of SUD during the 1 or 2 months before plan enrollment.

**Table 2.** Demographic and Clinical Information

Characteristic	Sample, No. %		*P* value for randomization check among plans
	Auto assigned	Entire state	
No.^a^	159 016	5 672 669	NA
Mean (SD) age, y	35.9 (12.7)	36.6 (13.2)	.91
Female	74 204 (46.7)	3 115 765 (54.9)	.001
Male	84 812 (53.3)	2 556 904 (45.1)	.001
Asian	8678 (5.5)	615 113 (10.8)	.10
Black	73 767 (46.4)	1 139 811 (20.1)	.36
White^b^	40 600 (25.5)	2 055 949 (36.2)	.71
Other or Hispanic^c^	35 971 (22.6)	1 861 796 (32.8)	.74
Supplemental income^d^	11 987 (7.2)	429 830 (7.6)	.95
Had substance use disorder claim(s) before assignment^e^	17 568 (11.0)	276 643 (4.9)^f^	.05

Abbreviations: NA, not applicable; SSI, Social Security income.

^a^ No. equals all unique members from 2009 to 2017.

^b^ White may include Hispanic and non-Hispanic.

^c^ Other race includes American Indian/Alaska Native, Hispanic-Puerto Rican, Unknown, and Other.^34^

^d^ Eligibility categories in addition to SSI for New York, New York were low-income family codes (47.2%), safety-net families (19.8%) and other (25.8%) (https://www.dropbox.com/s/wapa608vk9vs2od/aid_codes_pg_6.pdf?dl=0).

^e^ Percentage of members who had a substance use disorder claim during the 2 months before assignment.

^f^ The number only applies to the members who were assignable to a plan.

Table 2 also reports a randomization check. If autoassigned enrollees can be regarded as randomly assigned, there theoretically would be no significant differences among enrollees between plans. We regressed each baseline characteristic on the set of plan assignment indicators within the New York, New York sample. Thus, each row in the third column of Table 2 corresponds with a distinct regression, controlling for county-year-month of assignment fixed effects. We reported the *P* value from an *F* test that the plan effects were jointly different than 0, which is a test of the null hypothesis that the baseline characteristics do not differ significantly among recipients assigned to different plans, including in the frequency of eligibility by Social Security income. Eligibility for Medicaid was not strongly associated with Affordable Care Act–related expansions. A total of 136 144 (86%) of the beneficiaries in the study sample were eligible via codes appearing in all 3 waves of data. Significant imbalance appeared for the percentage of female participants and prior SUD indication. We examined plan means for these 2 variables by period and found small differences in the values across plans. For example, the range for female participants from 2012 to 2014 was within 6 percentage points, and the presence of SUD in claims for each period was within 2 percentage points. We do not expect an imbalance of this magnitude to interfere with the interpretation of plan effects.

In a descriptive analysis for context, Table 3 presents means for each indicator, grouped by category, for the autoassigned sample for the 3 periods and overall. Access to screening for SUD (period 1, 4.8%; 95% CI, 4.6%-5.1%; period 2, 8.2%; 95% CI, 7.9%-8.5%; period 3, 12.6%; 95% CI, 12.0%-13.3%), tobacco use (period 1, 0.9%; 95% CI, 0.8%-1.0%; period 2, 3.2%; 95% CI, 3.1%-3.3%; period 3, 4.9%; 95% CI, 4.6%-5.2%), and SUD diagnosis (period 1, 11.9%; 95% CI, 11.6%-12.2%; period 2, 15.1%; 95% CI, 14.9%-15.4%; period 3, 17.2%; 95% CI, 16.6%-17.7%) increased over time for the autoassigned samples. As indicated in Table 3, 14.2% (95% CI, 14.1%-14.4%) of the beneficiaries in the average plan had a claim indicating the presence of an SUD during the 6-month observation period. The characteristics of the members of the autoassigned groups changed over time, so the results in Table 3 cannot be interpreted as trends in plan effects (eTable 3 in the Supplement).

**Table 3.** Plan Performance on Quality of Substance Use Disorder Treatment in the Autoassigned Sample

Indicator	Mean (range), %			
	2009-2011	2012-2014	2015-2017	Aggregate
Access				
Screening and first assessment visits: outpatient care	4.8 (4.6-5.1)	8.2 (7.9-8.5)	12.6 (12.0-13.3)	7.6 (7.4-7.8)
Presence of an SUD diagnosis in claim	11.9 (11.6-12.2)	15.1 (14.9-15.4)	17.2 (16.6-17.7)	14.2 (14.1-14.4)
Any SUD service use for the first time	11.7 (11.4-12.0)	15.0 (14.8-15.2)	17.1 (16.5-17.6)	14.1 (13.9-14.3)
Tobacco screening	0.9 (0.8-1.0)	3.2 (3.1-3.3)	4.9 (4.6-5.2)	2.6 (2.5-2.7)
Patterns of care				
Follow-up after withdrawal (detoxification) management (14 d)	21.4 (18.7-24.0)	26.9 (25.0-28.8)	28.7 (24.9-32.5)	25.8 (24.3-27.2)
SUD Tx				
Initiation	47.1 (42.0-52.1)	46.9 (43.1-50.8)	42.3 (36.9-47.7)	45.9 (43.2-48.5)
Engagement	1.1 (0-2.1)	1.5 (0.6-2.5)	9.0 (5.8-12.1)	3.2 (2.2-4.1)
Engagement				
Alcohol pharmacotherapy	0	0.3 (−0.1-0.7)	0.4 (−0.2-1.0)	0.3 (0-0.5)
Opioid pharmacotherapy	61.4 (58.7-64.1)	62.1 (60.0-64.2)	41.5 (38.0-45.1)	58.1 (56.5-59.6)
Smoking cessation	1.6 (0.5-2.7)	0.8 (0.5-1.1)	7.2 (5.4-9.0)	2.2 (1.8-2.7)
SUD Tx engagement: follow-up within 30 d after ED visit	27.1 (19.7-34.5)	23.8 (19.8-27.9)	20.3 (15.8-24.8)	23.1 (20.3-25.9)
Identification of comorbidity with mental illness	14.2 (13.4-15.1)	20.1 (19.4-20.8)	24.5 (23.0-26.0)	19.0 (18.5-19.5)
Monthly expenditures on SUD Tx (mean-including zeros), $	141.2 (131.2-151.1)	268.5 (255.9-281.1)	680.4 (629.2-731.6)	272.5 (262.8-282.2)
% Of nonzero expenditures on SUD Tx	8.6 (8.3-8.8)	11.9 (11.6-12.1)	13.0 (12.5-13.5)	10.8 (10.7-11.0)
Receipt of psychosocial interventions	47.1 (45.9-48.3)	37.7 (36.9-38.6)	35.9 (34.2-37.5)	40.2 (39.6-40.9)
Outcomes				
Rapid readmission to inpatient SUD care 2-30 d postdischarge	23.5 (21.3-25.7)	27.8 (26.3-29.3)	30.5 (27.5-33.5)	27.1 (26.0-28.3)
Relapse (2-30 d)	11.0 (10.2-11.7)	13.6 (13.0-14.2)	33.2 (31.6-34.8)	15.6 (15.2-16.1)
Treatment continuation	3.1 (2.7-3.5)	3.6 (3.3-3.9)	14.2 (13.0-15.4)	5.0 (4.7-5.3)
Social connectedness (peer services)	0.5 (0.4-0.7)	0.6 (0.5-0.8)	0.5 (0.2-0.7)	0.6 (0.5-0.7)

Abbreviations: SUD, substance use disorder; Tx, treatment.

Table 4 summarizes the regression results. For 7 of the 18 indicators, an *F* test result revealed that plan effects were significant at the .05 level or better (eTable 4 in the Supplement). For individual plan results, positive numbers indicated better performance than average across all 9 plans, and negative numbers indicated worse performance. For overall performance in recognizing and treating SUD, some plans (eg, plan 3) performed systematically better than others. For access indicators, plans 3 and 5 performed 1% better than mean plan performance on identifying SUD, first-time service use, and tobacco screening, whereas plans 1 and 8 performed significantly worse for SUD diagnosis and service use. Indicators of SUD treatment were commonly interpreted as indicators of case-mix. With random assignment balancing characteristics, we could interpret them as the success of a plan in identifying individuals with SUD. For the process of care indicators, we found few significant differences (except for expenditures) compared with mean plan performance. The exceptions were plan 8 (lower follow-up rates after withdrawal management within 14 days and SUD treatment initiation [worse performance]), plan 4 (better identification of comorbid mental illness), and plan 5 (lower receipt of psychosocial interventions). Average monthly expenditures on SUD treatment varied between plans by roughly $100, with plans 6 and 3 spending significantly more on those treated (improved performance) and plans 1 and 7 spending significantly less (worse performance). Outcomes demonstrated minimal differences across plans, except for plan 4 (better performance in rapid readmission to inpatient SUD care postdischarge), plans 7 and 8 (lower relapse rates and better performance), and plan 8 (lower treatment continuation and worse performance).

**Table 4.** Regression of Plan Associations With Quality of Substance Use Disorder Treatment in the Autoassigned Sample

Indicator	Plan, %^a^									
	All (*P* value, *F* test)	1	2	3	4	5	6	7	8	9
Access										
Screening and first SUD assessment visits: outpatient care	.20	0	−0.1	−0.1	−0.3	−0.3	0.2	−0.1	0.4	1.1^b^
Presence of SUD diagnosis in claim	<.001^c^	−0.7^d^	0.4	1.0^c^	−0.3	1.1^c^	0.4	0.1	−1.0^c^	−0.8^b^
Any SUD service use for the first time	<.001^c^	−0.7^d^	0.4	1.0^c^	−0.3	1.1^c^	0.4	0.1	−1.0^c^	−0.7
Tobacco screening	<.001^c^	−0.2	0.2	0.5^c^	−0.1	0.6^c^	−0.3^d^	−0.1	−0.4^c^	0.0
Patterns of care										
Follow-up after withdrawal (detox) management, 14 d	.13	−0.1	−2.9	−1.8	−0.4	1.0	1.1	−1.1	8.1^d^	−1.5
SUD Tx										
Initiation	.01^b^	5.1	1.9	4.6	2.3	3.7	1.3	0.7	−15.2^c^	−9.1
Engagement	.59	1.4	1.0	0.9	0	0.3	−2.5	1.0	−1.9	0
Engagement										
Alcohol pharmacotherapy	.48	0.9^b^	0.2	−0.3	−0.2	−0.3	−0.3	−0.2	0.2	−0.3
Opioid pharmacotherapy	.76	−1.0	0.5	3.0	−1.2	−0.9	−0.7	2.1	−2.2	4.4
Smoking cessation	.44	−0.6	0.7	−1.2	−0.3	−0.1	−0.1	0.7	0.5	1.5
SUD Tx engagement: follow-up within 30 d of ED visit	.75	4.2	−3.6	4.6	−2.4	−3.4	0.3	−5.0	0.8	5.4
Identification of comorbid mental illness	.16	0.3	−0.5	0.6	1.7^d^	0.0	−0.5	−1.0	−0.7	−1.8
Monthly expenditures on SUD Tx (mean, including zeros), $	<.001^c^	−64.5^c^	−9.2	70.2^c^	−18.8	−13.1	123.9^c^	−68.5^c^	−16.1	44.2^b^
Receipt of psychosocial interventions	.11	−0.8	−0.3	0.8	1.2	−2.7^d^	1.1	−0.5	0.9	−0.6
Outcomes										
Rapid readmission to inpatient SUD care 2-30 d postdischarge	.05	−0.5	−3.8^b^	0.9	3.8^d^	0.7	0.0	−3.6	0.0	1.6
Relapse (2-30 d)	<.001	−0.8	−0.7	1.8^b^	1.4^b^	−0.5	1.6^b^	−2.5^d^	−2.2^d^	2.0
Treatment continuation	.004	0	0	0.9	0.1	0.3	0.6	−0.7	−1.6^c^	0.6
Social connectedness (peer services)	.98	0	0	0.1	0.1	0	−0.1	0.1	−0.1	0

Abbreviations: SUD, substance use disorder; Tx, treatment.

^a^ Positive numbers reflect better performance than the average performance across all 9 plans, and negative numbers indicate worse performance. Percentage of expenditures are not included (19th indicator).

^b^ *P* < .05.

^c^ *P* < .001.

^d^ *P* < .01.

## Discussion

Medicaid is the largest single source of health coverage in the US^35^ and NYS. Although NYS Medicaid reports standardized indicators and metrics, few are designed to monitor performance and inform plan choice.^29,30,31^ This study developed a set of algorithms to monitor and provide accountability that could be applied to other Medicaid settings. The study results potentially provide an initial step toward making valid comparisons of SUD treatment across MMC plans in the largest market in the country, the 5 boroughs of New York, New York. Overall, differences in the processes of care were relatively modest, except for differences in spending. This suggested that improved performance cannot rely on redistributing recipients across plans but instead requires system-wide improvements.

Recognition of health problems is the foundation of effective health care. The 2017 National Survey on Drug Use and Health indicated that 7.6% of adults nationwide met *Diagnostic and Statistical Manual of Mental Disorders* (Fourth Edition) criteria for SUD during the prior year.^36^ Within the group of autoassignees, the New York, New York MMC plans recognized SUD at a higher rate than the national average (17.16% as of 2015-2017), but this may be because of systematic differences among the populations compared. In addition, New York, New York introduced targeted policies, such as the Thrive Initiative on mental health and substance use,^37^ that may have been associated with increased SUD identification rates.

Effective treatment engagement is necessary after an SUD is recognized.^38,39^ This requires substantial outreach and follow-up. According to the 2017 National Survey on Drug Use and Health,^40^ of almost 21 million people meeting symptom criteria for being in need of treatment for SUD, 96% reported that they did not feel that they needed treatment of any kind (including nonprofessional peer support groups, therapy, and medications), signaling the magnitude of the challenge. Unsurprisingly, the present analyses found low treatment engagement, including on indicators of 2 or more visits after a diagnosis, engagement in smoking cessation, and pharmacotherapy for alcohol use disorder, which were consistent with the literature.^41,42,43^ Engagement in opioid use disorder pharmacotherapy was the only indicator to surpass 50%, at 58% on average from 2009 to 2017. Most indicators were stable at a low level (less than 50%), including many processes of care measures,^29^ such as SUD treatment engagement, engagement in alcohol pharmacotherapy, smoking cessation engagement, and any expenditure for SUD treatment. This might be associated with mandatory inclusion of prescription drugs for opioid use disorder in MMC plan formularies and allowances of increased costs.^44^ To further encourage medication use, plans could remove logistical barriers (eg, prior authorization) and cover structured peer support and training.^45^

Care is limited by available funds. During the past 40 years nationally, total treatment expenditures covered by Medicaid have increased substantially for mental health, but not as much for SUD.^46,47^ Medicaid expansion has increased the coverage and payment for admissions to specialty facilities and increased prescriptions for SUD medications.^48^ However, most states do not cover all recommended levels of care.^49^ Outpatient care has centered around SUD medication vs psychotherapy or recovery services, with reimbursement rates remaining lower than for other private or public insurance programs.^50^ Furthermore, plans have little incentive to provide good care that might reduce costs downstream,^51^ partially because of the high churn in Medicaid populations, especially those with SUD.^52^

Treatment of SUD is generally cost-effective at a full dose. A $1 investment in methadone treatment can generate a $4 to $5 return in reduced health care expenditures.^53^ People with treated alcohol use disorder utilize health care at half the rate and generate costs that are half as much as those of untreated individuals.^53^ This may imply that risk-bearing MMC plans should be eager to finance SUD treatment. However, high plan-level turnover in Medicaid limits an ability to recoup savings, suggesting the need to incentivize prevention and treatment contractually.

Absence of a consensus about quality measures limits the effectiveness of payer and plan monitoring. Section 1115 waivers and independent Medicaid designs across states hinder consolidated reporting and comparison measurements.^54,55^ Infrastructure problems, such as lack of electronic health record adoption,^56^ limit the viability of hybrid SUD quality measures using claims and electronic health record data.

Starting in 2024, the SUPPORT Act of 2018 requires all states to report the Behavioral Health Services Core Set of quality measures, representing an opportunity to evaluate indicators to include. Disease recurrence, one indicator, is not fully captured in claims, missing those who experienced relapse and did not get medical help.^57^ Mortality is the ultimate outcome but may occur well after poor care is provided and requires linking to non-Medicaid data sets.

Good quality SUD care must be supported by regulation and adequate financing. The Affordable Care Act requires coverage parity. However, full parity requires structuring payments to encourage serving enrollees with behavioral health issues.^58^ This can be inhibited by limited recognition of diagnoses in risk-adjusted plan payment algorithms.^59^ In the absence of full parity, capitated managed care plans, which are not limited to MMC plans, have incentives to set higher barriers for treatment for SUDs and behavioral health problems.^60,61^

### Limitations

To ensure that our comparisons could be interpreted as plan effects, the sample was limited to a small subset of the Medicaid population of New York, New York. Although the autoassigned sample had similar demographic characteristics, there are likely unmeasured differences with the full Medicaid population. Balancing this limitation (similar to randomized clinical trials) is that random assignment offers the opportunity to avoid differences in case-mix across plans that would overcome the potential equivalence in Medicaid populations across plans that is needed to fairly compare their performance. To construct the indicators, we required plan enrollment of at least 6 months, which does not hold for all enrollees. We are aware that health services claim data cannot provide nuanced clinical and social information about clients, thus interfering with nuanced interpretations of patterns of care and outcomes.

Changes in measured mean performance over time could be because of factors other than MMC plan performance. Autoassigned population characteristics changed during the study period. Changes in codes and coding practices across periods mean that we cannot interpret trends across time. However, within any period, the study design allowed us to make statements about relative plan performance.

## Conclusions

This cross-sectional study highlights the importance of monitoring access, quality, and outcomes of SUD treatment among Medicaid-covered populations. Greater investment in client engagement and the financial means to hire sufficient care coordinators^62,63^ are useful steps. Rewarding plans for effective treatment and clinical outcomes might incentivize strategies to engage and retain patients. There is value in leveraging autoassignment to understand the extent of true plan performance differences so that states (and researchers) can learn from high (and low) performers. This could ensure that pay-for-performance incentives flow to plans because of performance rather than case-mix. Valid plan comparisons could also assist in state-plan contracting decisions.

## Notes

Supplement.
eMethods.
**eTable 1. **Algorithms of indicators **eTable 2. **SUD population identification **eTable 3. **Plan performance on quality of substance use disorder treatment (full sample) **eTable 4. **Regression—plan effects on quality of SUD treatment (full sample) **eTable 5. **STROBE statement—checklist of items that should be included in reports of cross-sectional studies
