# Supplementary material for: Performance Metrics of Substance Use Disorder Care Among Medicaid Enrollees in New York, New York
Source: JAMA Health Forum. 2022 Jul 1;3(7):e221771. doi: 10.1001/jamahealthforum.2022.1771 (PMC9250047; doi:10.1001/jamahealthforum.2022.1771)
Supplement: Supplement. — eMethods. eTable 1. Algorithms of indicators eTable 2. SUD population identification eTable 3. Plan performance on quality of substance use disorder treatment (full sample) eTable 4. Regression—plan effects on quality of SUD treatment (full sample) eTable 5. STROBE statement—checklist of items that should be included in reports of cross-sectional studies [file jamahealthforum-e221771-s001.pdf]

Supplemental Online Content

Alegría M, Falgas-Bague I, Fukuda M, et al. Performance metrics of substance use disorder care among Medicaid enrollees in New York, New York. JAMA Health Forum. 2022;3(7):e221771.
doi:10.1001/jamahealthforum.2022.1771

eMethods.
eTable 1. Algorithms of indicators
eTable 2. SUD population identification
eTable 3. Plan performance on quality of substance use disorder treatment (full sample)
eTable 4. Regression—plan effects on quality of SUD treatment (full sample)
eTable 5. STROBE statement—checklist of items that should be included in reports of cross-sectional studies

This supplemental material has been provided by the authors to give readers additional information about their work.

© 2022 Alegría M et al. *JAMA Health Forum.*

eMethods.

Data Cleaning

A merger of two plans occurred in our second data period (year withheld for confidentiality reasons). Our data attributed enrollees to the acquiring plan throughout all data periods. In the case of another plan acquired during our last data period, we are able to attribute enrollees to the plan before it was acquired. In other words, this plan is observed separately while it was independent, but once acquired, enrollees are attributed to the acquiring plan.

Selection of Performance Indicators

Using Donabedian's conceptual framework, we focused on structure, process, and outcomes in examining health services and evaluating the quality in MMC. Specifically, we examined (a) structure – measures relating to treatment by MMC plan; (b) process – measures relating to treatment patterns of care, including types of treatments received (pharmacological care); and (c) outcomes – measures relating to events like relapse in treatment. The selection of the performance indicators underwent extensive review to ensure evidence supported use of the performance algorithms for data analyses. This required a thorough review of the procedure, diagnosis, and drug codes used in identifying services, treatments, and medications used in SUD care. Co-investigators with specialized SUD treatment expertise reviewed all SUD performance indicators, inclusion criteria, measures, and codes to ensure algorithms were accurately constructed.

Indicator Refinement Process

The research team grouped indicators into categories of SUD treatment access, process measures of patterns of care, and outcomes. They selected performance measures from the scientific literature as well as based on metrics defined in the Healthcare Effectiveness Data Information Set (HEDIS). Experts in the areas of behavioral health and the research team detailed and reviewed sample inclusion criteria, measures, claims data codes and definitions, and research references. Based on an extensive review, indicators were finalized.

During consultation with coinvestigators with specialized substance use treatment expertise, the issues below were identified and resolved.

Changes in treatment definitions over time. Given the nine years of data (2009-2017), the team reviewed changes in HEDIS, procedural codes, and diagnostic codes for algorithms. Instead of determining separate algorithms for each year of data, the team elected to use the most recent HEDIS definitions, procedural codes, and diagnostic codes to inform the algorithms. The one exception to this rule was psychosocial interventions given the expanded use and coverage of counseling for SUD in Medicaid. For these interventions, procedural and diagnostic code changes over the data period were captured and applied to these algorithms.

ICD codes. Most of the data contained ICD-9 codes but the team developed a crosswalk to incorporate updates and to capture ICD-10 codes in the more recent data.

Procedural codes. Clinicians reviewed all Clinical Procedural Terminology (CPT) codes and Healthcare Common Procedure Coding System (HCPCS) codes used in the algorithms; codes that could not accurately define SUD treatment or diagnosis were removed to ensure a conservative approach.

Medication lists for SUD treatment. These were created with codes generated from existing literature, checked against the National Drug Code (NDC) list, and proprietary and non-proprietary names and codes generated

through the Food and Drug Administration web converter tool. Clinicians and pharmacology experts reviewed and flagged codes used for non-SUD diagnoses, prescribed as off-label use, or considered inappropriate, including formulations of medications used specifically for surgical treatment. These codes were then dropped from the algorithms. For NDC codes used in treatment and identification of SUD, specialists reviewed all drug codes used to identify opioid use disorder, alcohol use disorder, nicotine addiction, and cocaine addiction. A researcher specializing in addiction and pain management performed an additional review to remove drugs used in surgery and pain management. A co-investigator with tobacco cessation expertise reviewed all tobacco use disorder and treatment indicators as well as HCPCS, ICD-9 and ICD-10, CPT, and NDC codes. Bupropion was identified as a potentially confounding medication given its use in other applications, so a decision was made to analyze treatment with and without bupropion.

Billing and procedural codes. The director of billing compliance at Massachusetts General Hospital reviewed all billing and procedural codes.

HEDIS. One change in the algorithms involving HEDIS restrictions pertains to the continuous enrollment requirements for SUD treatment. The definition of continuous enrollment is: 60 days prior to the Index Episode Start Date (IESD) through 48 days after IESD (109 total days) for SUD service use and treatment initiation and engagement; and 30 days prior to the IESD through 30 days after the IESD for initiation and engagement in pharmacotherapy (61 total days). Given the impacts of the restriction on the randomized sample, the analysis team decided to conduct the analyses both with and without continuous enrollment.

Accommodating Algorithms to the Dataset

Our sample includes auto-assigned enrollees in MMC plans in New York City (NYC). Auto-assigned members are those who do not actively select a plan and are then randomly assigned to a plan. Enrollees are aged 18 to 64 years and the sample excludes dually eligible members, those whose family members are enrolled in MMC at time of assignment, and those enrolled in a MMC plan within a year prior to assignment. We identified the plans covering those enrollees and included all the plan's enrollees in the auto-assignee sample analysis. While most plan enrollees are from NYC, this process also captured individuals enrolled in these plans who live outside of NYC. Recipients in auto-assigned plans not in operation during the study period were excluded. The research team operationalized algorithms by adjusting the definition of population to include auto- assigned enrollees who were enrolled in Medicaid for at least 6 consecutive months, starting in 2009-2017. We also adjusted the time frame of the algorithms to only keep a member's first eligible enrollment episode.

© 2022 Alegría M et al. *JAMA Health Forum.*

eTable 1. Algorithms of Indicators

The complete Value Set Directory and National Drug Directory mentioned in the Algorithms of Indicators are available from the authors. Directories are not included here because the total are exceeding 200 pages.

Access

Indicator	Screening and 1st assessment visits - outpatient care	Notes
**Sample inclusion criteria**		
Age	18-64	
Enrollment time	Enrolled in current month with at least one outpatient visit during **the measurement period**	
SUD condition	No	
**Measure**		
Numerator	Members whose first outpatient claim in the first six months of their eligible enrollment episode included an SUD screening or SUD assessment.	
Denominator	Members who had an outpatient claim in the first six months of their eligible enrollment episode.	
**References**		
	Rieckmann T, Renfro S, McCarty D, Baker R, McConnell KJ. Quality Metrics and Systems Transformation: Are We Advancing Alcohol and Drug Screening in Primary Care?. Health Serv Res. 2018;53(3):1702-1726. doi:10.1111/1475-6773.12716	
	Porter RR, Hanson BL, Smith O. Medicaid Policies for Alcohol SBI Reimbursement 2017. https://scholarworks.alaska.edu/bitstream/handle/11122/11974/2017_08_31-MedicaidPoliciesAlcoholSBIReimbursement.pdf?sequence=1&isAllowed=y Accessed April 24, 2022	
	Anderson ER. Reimbursement in Teleneurology. In: Tsao JW, Demaerschalk BM, eds. Teleneurology in Practice: A Comprehensive Clinical Guide. Springer; 2015:183-187.	

	Fussell HE, Rieckmann TR, Quick MB. Medicaid reimbursement for screening and brief intervention for substance misuse. Psychiatr Serv. 2011;62(3):306-309.	
**Claims data code:**		
Identification of screening and first assessment visit		
ICD-9 Codes	V79.1 Patient completed a standardized health risk assessment tool for risky alcohol or drug use	Rieckmann 2017
	V82.9 Patient completed a standardized health risk assessment tool following a positive brief screening for risky alcohol or drug use	Rieckmann 2017
Original Codes	Corresponding ICD-10 codes	
ICD-9 Conversion V79.1 Patient completed a standardized health risk assessment tool for risky alcohol or drug use	Z13.89 Encounter for screening for other disorder	www.10icddata.com & www.aapc.com/icd-10/codes
ICD-9 Conversion V82.9 Patient completed a standardized health risk assessment tool following a positive brief screening for risky alcohol or drug use	Z13.9 Encounter for screening, unspecified	www.10icddata.com & www.aapc.com/icd-10/codes
CPT Codes	90791 (Psychiatric diagnostic interview examination)	Anderson 2015
	90832-90834 and 90836-90838 (Individual psychotherapy)	Anderson 2015
	99201-99205 (Office or outpatient telemedicine visits)	Anderson 2015
	99420 with an ICD-9 V79.1 or V82.9 Patient completed a standardized health risk assessment tool following a positive brief screening for risky alcohol or drug use	Rieckmann 2017
	99408 Patient completed a standardized health risk assessment tool and received a 15-to-29 minute intervention	Rieckmann 2017

	99409 Patient completed a standardized health risk assessment tool and received a 30 minute or longer intervention	Rieckmann 2017
HCPCS Codes	H0002 Alcohol and/or drug screening to determine eligibility for Tx program	Porter 2017
	H0004 Behavioral health counseling and therapy per 15 minute (alcohol and/or drug services)	Porter 2017
	H0049, H0047 Alcohol and/or other drug abuse services, not otherwise specified	Fusell 2011
	H0050	Fusell 2011
	4214 (NY specific OASAS code for SUD assessment)	
**Indicator**	**Presence of a Substance Use Disorder (SUD) diagnosis in claim (ASAM)**	**Notes**
**Sample inclusion criteria**		
Age	18-64	
Enrollment time	Enrolled at least one month in a given calendar year	
SUD condition	No restriction	
**Measure**		
Binary indicator	Equals "1" if an enrollee had a at least one SUD Dx, or had at least one prescription for medications commonly used to treat addiction, or was administered to detoxification or rehabilitation procedures specifically for alcohol or drug disorder treatment in any outpatient, inpatient, or ED claims in that given year, and "0" otherwise.	
Numerator	Members who had at least one SUD Dx, or had at least one prescription for medications commonly used to treat addiction, or were administered detoxification or rehabilitation procedures specifically for alcohol or drug disorder treatment in any outpatient, inpatient, or ED claims.	
Denominator	All members in the underlying sample.	
**References**		
	American Society of Addiction Medicine. The ASAM Performance Measures. Chevy Chase, MD: American Society of Addiction Medicine (ASAM);2014.	

	Clark RE, Samnaliev M, McGovern MP. Treatment for co-occurring mental and substance use disorders in five state Medicaid programs. Psychiatr Serv. 2007;58(7):942-948. *	Used to define SUD and treatment
	Harris AH, Weisner CM, Chalk M, Capoccia V, Chen C, Thomas CP. Specifying and Pilot Testing Quality Measures for the American Society of Addiction Medicine's Standards of Care. J Addict Med. 2016;10(3):148-155.	Use for AUD and OUD Dx and Detox Identification, provides both ICD-9 and 10 codes
	Mattke S, Predmore Z, Sloss E, Wilks A, Watkins KE. Evidence for Misspecification of a Nationally Used Quality Measure for Substance Use Treatment. J Healthc Qual. 2018;40(4):228-235.	Use for AUD and OUD Medication Treatment
	Ross S, Peselow E. Pharmacotherapy of addictive disorders. Clin Neuropharmacol. 2009;32(5):277-289.	Use for NUD and Cocaine Medication Treatment
	Garnick DW, Horgan CM, Chalk M. Performance measures for alcohol and other drug services. Alcohol Research and Health. 2006;29:19–26	Justifies use of HCPCS
**Claims data codes:**		
ICD-9/10 codes for SUD Dx	See value set "Comprehensive SUD Dx"	
Medications		
Prescription	See "Med for OUD", "Med for AUD", “Med for Nicotine” and "Med for Cocaine” in the NDC directory	
Injectable or Service	Injectable naltrexone was identified based on a HCPCS code (J2315) in the medical claims. Buprenorphine service or implant can be identified with HCPCS codes J0570, J0571, J0572, J0573, J0574, J0575, and J0592. As methadone may only be used for MAT through in-office dispensing in licensed treatment centers, the methadone (OTP) service was also identified based on a HCPCS code (H0020 and S0109).	
Detoxification or rehabilitation procedures specifically for alcohol or drug disorder treatment	See value set "Detoxification"	

Other SUD specific service use	See value set "AOD procedures"	
Notes	Source of the code is included in the value set document	
	Based on Harris et al. (2011) and (2016): the Performance Measures Panel included all ICD alcohol abuse and dependence codes, except in-remission diagnoses. The rationale for excluding in-remission codes is that those are intended to signify that a patient's symptoms are not in need of current treatment. (American Psychiatric Association, 2013).	
**Indicator**	**Any SUD service use for the 1st time**	**Notes**
**Sample inclusion criteria**		
Age	18-64	
Continuous enrollment time	One month prior to the IESD through one month after the IESD. See the IESD definition below.	
SUD condition	New episode of SUD during the Intake Period	
**Measure**		
Binary indicator	A binary indicator of whether a claim is counted as the 1st time of any SUD service use	
Date of 1st SUD service use	The date of the 1st time service use	
Numerator	Members who have an SUD claim in the first six months of their eligible enrollment episode and are continuously enrolled in Medicaid for two months prior and one month after (continuous enrollment time).	
Denominator	All members in the underlying sample	
How to operationalize	First, find all claims in which the member received SUD service use; second, sort by the service date; finally, the earliest date is the date for 1st time service use.	
**References**		
	National Committee for Quality Assurance. Proposed new measures for hedis 2017: Follow-up after emergency department visit for mental illness and alcohol and other drug dependence. 2016	

	HEDIS for the Quality Rating System Value Set Directory_2016-2018	
	Clark RE, Samnaliev M, McGovern MP. Treatment for co-occurring mental and substance use disorders in five state Medicaid programs. Psychiatr Serv. 2007;58(7):942-948.	Used to define SUD and treatment
	Callaghan RC, Cunningham JK, Allebeck P, Arenovich T, Sajeev G, Remington G, et al: Methamphetamine use and schizophrenia: a population-based cohort study in California. Am J Psychiatry 2012;169(4):389–396.	
	Harris AH, Weisner CM, Chalk M, Capoccia V, Chen C, Thomas CP. Specifying and Pilot Testing Quality Measures for the American Society of Addiction Medicine's Standards of Care. J Addict Med. 2016;10(3):148-155.	Used for AUD and OUD Dx and Detox Identification, provides both ICD-9 and 10 codes
	Mattke S, Predmore Z, Sloss E, Wilks A, Watkins KE. Evidence for Misspecification of a Nationally Used Quality Measure for Substance Use Treatment. J Healthc Qual. 2018;40(4):228-235.	Used for AUD and OUD Medication Treatment
	Ross S, Peselow E. Pharmacotherapy of addictive disorders. Clin Neuropharmacol. 2009;32(5):277-289.	Used for NUD and Cocaine Medication Treatment
	Garnick DW, Horgan CM, Chalk M. Performance measures for alcohol and other drug services.<br>Alcohol Research and Health. 2006;29:19–26	Justifies use of HCPCS
	Centers for Medicare & Medicaid Services. *Core Set of Adult Health Care Quality Measures for Medicaid (Adult Core Set): Technical Specifications and Resource Manual for Federal Fiscal year 2018 Reporting.* Accessed April 25, 2022. Available at https://www.medicaid.gov/medicaid/quality-of-care/downloads/medicaid-adult-core-set-manual.pdf	
**Claims data code:**		
SUD service use	Inpatient, intensive outpatient, partial hospitalization, outpatient, telehealth, detoxification, or ED visit during the Intake period with a diagnosis of SUD abuse or dependence (SUD Dx), or filled prescription for medications commonly used to	Source: HEDIS measure specifications (2018) QRS Measure Technical Specifications

	treat addition, or was administered detoxification or rehabilitation procedures specifically for alcohol or drug disorder treatment.	
ICD-9/10 codes for SUD Dx	See value set "Comprehensive SUD Dx"	
Medications		
Prescription	See "Med for OUD", "Med for AUD", "Med for Nicotine" and "Med for Cocaine" in the NDC directory	
Injectable or Service	Injectable naltrexone was identified based on a HCPCS code (J2315) in the medical claims. Buprenorphine service or implant can be identified with HCPCS codes J0570, J0571, J0572, J0573, J0574, J0575, and J0592. As methadone may only be used for MAT through in-office dispensing in licensed treatment centers, the methadone (OTP) service was also identified based on a HCPCS code (H0020 and S0109).	
Detoxification or rehabilitation procedures specifically for alcohol or drug disorder treatment	See value set "Detoxification"	
Other SUD specific service use	See value set "AOD procedures"	
Notes	Source of the code is included in the value set document	
**Indicator**	**Tobacco Screening**	**Notes**
**Sample inclusion criteria**		
Age	18-64	
Enrollment time	Enrolled at least one month in a given calendar year	
SUD condition	All	
**Measure**		
Numerator	Members who also received a tobacco screen in the first six months of their eligible enrollment episode.	

Denominator	All members in the underlying sample	
**References**		
	American Lung Association. Billing Guide for Tobacco Screening and Cessation. 2018	Kept ALA codes that indicated that the patient was currently a smoker and omitted preventative and second-hand smoke references.
**Claims data code:**		
	See value set "Tobacco Screening Treatment"	

Patterns of Care

Indicator	Follow-up after Withdrawal (Detoxification) Management	Notes
**Sample inclusion criteria**		
Age	18-64	
Enrollment time	Enrolled at least one month in a given calendar year	
SUD condition	Had SUD withdrawal management episodes at any time in the enrolled year	
**Measure**		
Numerator (7 days)	Members in the denominator who also had a follow-up treatment in the first six months of their eligible enrollment episode. To be an appropriate follow-up treatment to a withdrawal management episode, the service or prescription must have taken place between 1 and 7 days after discharge, have an SUD diagnosis, and not represent a new withdrawal management episode.	
Numerator (14 days)	Members in the denominator who also had a follow-up treatment in the first six months of their eligible enrollment episode. To be an appropriate follow-up treatment to a withdrawal management episode, the service or prescription must have taken place between 1 and 14 days after discharge, have an SUD diagnosis, and not represent a new withdrawal management episode.	
Denominator	Members who had a withdrawal management episode in the first six months of their eligible enrollment episode. Withdrawal management episodes are defined such that withdrawal treatments within 3 days are grouped together in the same episode.	
How to operationalize	Create a binary indicator for each eligible withdrawal management episode whether there is a follow-up service or prescription as defined in the numerator. Then calculate the mean or % of 1s per plan per year.	
**References**		
	Thomas CP, Ritter GA, Harris AHS, Garnick DW, Freedman KI, Herbert B. Applying American Society of Addiction Medicine Performance Measures in Commercial Health Insurance and Services Data. J Addict Med. 2018;12(4):287-294.	Definition of the measure

	Harris AH, Weisner CM, Chalk M, Capoccia V, Chen C, Thomas CP. Specifying and Pilot Testing Quality Measures for the American Society of Addiction Medicine's Standards of Care. J Addict Med. 2016;10(3):148-155.	Use for AUD and OUD Medication Treatment
	Mattke S, Predmore Z, Sloss E, Wilks A, Watkins KE. Evidence for Misspecification of a Nationally Used Quality Measure for Substance Use Treatment. J Healthc Qual. 2018;40(4):228-235.	Use for AUD and OUD Medication Treatment
	Ross S, Peselow E. Pharmacotherapy of addictive disorders. Clin Neuropharmacol. 2009;32(5):277-289.	Use for NUD and Cocaine Medication Treatment
**Claims data code:**		
Withdrawal management episodes	Withdrawal management episodes were identified by daily procedure code and were bundled so that no episode had a gap in treatment greater than 3 days (i.e., treatments within 3 days are grouped together in the same episode). See value set "Withdrawal Management Episode".	Source: Thomas et al. (2018)
Follow-up treatment to a withdrawal management episode	To be an appropriate follow-up treatment to a withdrawal management episode, the service or prescription must have taken place between 1 and 7 days after discharge (based on the discharge date or the date when withdrawal management ends), have an SUD diagnosis (not restricted to the original reason for withdrawal management), and not represent a new withdrawal management episode. Additional claims or prescriptions on the last day of the episode did not count as follow-up. Various types of follow-ups were examined (i.e., AUD or OUD pharmacotherapy in the absence of follow-up visits). See below for the identifications of follow-up services and prescriptions.	
Inpatient follow-up post-withdrawal management	Revenue codes for rehabilitation room and board: 0124, 0128, 0190, 1002	Source: Thomas et al. (2018)
Outpatients follow-up post-withdrawal management	CPT4 codes: 99221–99239 with SUD ICD-9 diagnosis	
	All visits or services associated with SUD diagnosis codes that are not identified as inpatient	Source: Thomas et al. (2018)

Prescription for SUD	See "Med for OUD", "Med for AUD", "Med for Nicotine" and "Med for Cocaine" in the NDC directory	Source: Harris et al. (2016), Thomas et al. (2018)
	Injectable naltrexone was identified based on a HCPCS code (J2315) in the medical claims. Buprenorphine service or implant can be identified with HCPCS codes J0570, J0571, J0572, J0573, J0574, J0575, and J0592.As methadone may only be used for MAT through in-office dispensing in licensed treatment centers, the methadone (OTP) service was also identified based on a HCPCS code (H0020 and S0109).	
**Indicator**	**SUD Treatment Initiation**	**Notes**
**Sample inclusion criteria**		
Age	18-64	
Intake period	January 1 – November 15 of the measurement year. The Intake period is used to capture new episodes.	
Continuous enrollment time	One month prior to the IESD through one month after the IESD. See the IESD definition below.<br>Build the indicator in two ways: with and without this continuous enrollment restriction.	IESD: The earliest date of service. See more details below.
SUD condition	New episode of SUD during the Intake period	
**Measure**		
**Note**	This algorithm comes in two versions – with and without the continuous enrollment requirement.	
**Numerator**	**# of eligible enrollees (in the denominator) who initiated SUD Treatment within 14 days of the diagnosis.**	
	Initiation of SUD treatment through an inpatient admission, outpatient visit, intensive outpatient encounter, or partial hospitalization within 14 days of diagnosis.	

	If the Index Episode was an inpatient discharge, the inpatient stay is considered initiation of treatment and the member is compliant.	
	If the Index Episode was an outpatient, intensive outpatient, partial hospitalization, telehealth, detoxification, or ED visit, the member must have an inpatient admission, outpatient visit, telehealth, intensive outpatient encounter, or partial hospitalization, with a diagnosis of SUD abuse or dependence, on the Index Episode Start Date (IESD) or in the 13 days after the IESD (14 total days). If the IESD and the initiation visit occur on the same day, they must be with different providers in order to count. Any of the following code combinations meet criteria:	
	• An acute or nonacute inpatient admission with a diagnosis matching the IESD diagnosis cohort using one of the following: Alcohol Abuse and Dependence Value Set, Opioid Abuse and Dependence Value Set, Other Drug Abuse and Dependence Value Set. To identify acute and nonacute inpatient admissions:	
	1. Identify all acute and nonacute inpatient stays (Inpatient Stay Value Set).	
	2. Identify the admission date for the stay.	
	• IET Stand Alone Visits Value Set with a diagnosis matching the IESD diagnosis cohort using one of the following: Alcohol Abuse and Dependence Value Set, Opioid Abuse and Dependence Value Set, Other Drug Abuse and Dependence Value Set, with or without a telehealth modifier (Telehealth Modifier Value Set).	
	• IET Visits Group 1 Value Set with IET POS Group 1 Value Set and a diagnosis matching the IESD diagnosis cohort using one of the following: Alcohol Abuse and Dependence Value Set, Opioid Abuse and Dependence Value Set, Other Drug Abuse and Dependence Value Set with or without a telehealth modifier (Telehealth Modifier Value Set).	

	• IET Visits Group 2 Value Set with IET POS Group 2 Value Set and a diagnosis matching the IESD diagnosis cohort using one of the following: Alcohol Abuse and Dependence Value Set, Opioid Abuse and Dependence Value Set, Other Drug Abuse and Dependence Value Set with or without a telehealth modifier (Telehealth Modifier Value Set). A telephone visit (Telephone Visit Value Set) with a diagnosis matching the IESD diagnosis cohort using one of the following: Alcohol Abuse and Dependence Value Set, Opioid Abuse and Dependence Value Set, Other Drug Abuse and Dependence Value Set.	
	• An online assessment (Online Assessment Value) set with a diagnosis matching the IESD diagnosis cohort using one of the following: Alcohol Abuse and Dependence Value Set, Opioid Abuse and Dependence Value Set, Other Drug abuse and Dependence Value Set.	
	• If the Index Episode was for a diagnosis of alcohol abuse or dependence (Alcohol Abuse and Dependence Value Set) a MAT dispensing event (MAT for Alcohol Abuse or Dependence Medications List; MAT for Opioid Abuse or Dependence Medications List) or a claim for MAT (Medication Assisted Treatment Value Set).	
	• If the Index Episode was for a diagnosis of opioid abuse or dependence (Opioid Abuse and Dependence Value Set) a MAT dispensing event (MAT for Alcohol Abuse or Dependence Medications List; MAT for Opioid Abuse or Dependence Medications List) or a claim for MAT (Medication Assisted Treatment Value Set).	
	Note: If a member is compliant for the Initiation numerator for any diagnosis cohort (i.e., alcohol, opioid, other drug), count the member once in the Total Initiation numerator. If the member is compliant for multiple cohorts, only count the member once in the Total Initiation Numerator. The “Total” column is not the sum of the diagnosis columns. Exclude the member from the denominator for both indicators (Initiation of SUD Treatment and Engagement of SUD Treatment)	

	if the initiation of treatment event is an inpatient stay with a discharge date after December 1 of the measurement year.	
**Denominator**	Have SUD Dx code combination (provided by MGH to identify specific kinds of SUD claims) on the first SUD claim in the first six months of their eligible enrollment episode; AND have a 'negative diagnosis history' AND meet the continuous enrollment requirement.<br>Follow the steps below.	
	**Step 1**<br>Identify the Index Episode. Identify all members in the specified age range who during the Intake Period had one of the following:	
	• An outpatient visit, telehealth, intensive outpatient visit or partial hospitalization with a diagnosis of SUD abuse or dependence. Any of the following code combinations meet criteria:	
	– IET Stand Alone Visits Value Set with one of the following: Alcohol Abuse and Dependence Value Set, Opioid Abuse and Dependence Value Set, Other Drug Abuse and Dependence Value Set, with or without a telehealth modifier (Telehealth Modifier Value Set).	
	– IET Visits Group 1 Value Set with IET POS Group 1 Value Set and with one of the following: Alcohol Abuse and Dependence Value Set, Opioid Abuse and Dependence Value Set, Other Drug Abuse and Dependence Value Set, with or without a telehealth modifier (Telehealth Modifier Value Set).	
	– IET Visits Group 2 Value Set with IET POS Group 2 Value Set and with one of the following: Alcohol Abuse and Dependence Value Set, Opioid Abuse and Dependence Value Set, Other Drug Abuse and Dependence Value Set, with or without a telehealth modifier (Telehealth Modifier Value Set).	
	• A detoxification visit (Detoxification Value Set) with one of the following: Alcohol Abuse and Dependence Value Set, Opioid Abuse and Dependence Value Set, Other Drug Abuse and Dependence Value Set.	

	• An ED visit (ED Value Set) with one of the following: Alcohol Abuse and Dependence Value Set, Opioid Abuse and Dependence Value Set, Other Drug Abuse and Dependence Value Set.	
	• An acute or nonacute inpatient discharge with one of the following: Alcohol Abuse and Dependence Value Set, Opioid Abuse and Dependence Value Set, Other Drug Abuse and Dependence Value Set. To identify acute and nonacute inpatient discharges:	
	1. Identify all acute and nonacute inpatient stays (Inpatient Stay Value Set).	
	2. Identify the discharge date for the stay.	
	• A telephone visit (Telephone Visits Value Set) with one of the following: Alcohol Abuse and Dependence Value Set, Opioid Abuse and Dependence Value Set, Other Drug Abuse and Dependence Value Set.	
	• An online assessment (Online Assessments Value Set) with one of the following: Alcohol Abuse and Dependence Value Set, Opioid Abuse and Dependence Value Set, Other Drug Abuse and Dependence Value Set.	
	Note: For members with more than one episode of SUD abuse or dependence, use the first episode. For members whose first episode was an ED visit that resulted in an inpatient stay, use the inpatient discharge. When an ED visit and an inpatient stay are billed on separate claims, the visit results in an inpatient stay when the admission date for the inpatient stay occurs on the ED date of service or one calendar day after. An ED visit billed on the same claim as an inpatient stay is considered a visit that resulted in an inpatient stay.	
	**Step 2**<br>Select the Index Episode and stratify based on age and SUD diagnosis cohort.	
	If the member has a claim with a diagnosis of alcohol abuse or dependence (Alcohol Abuse and Dependence Value Set), place the member in the alcohol cohort. If the member has a diagnosis of opioid abuse of dependence (Opioid Abuse and Dependence Value Set), place the member in the opioid cohort. If the member has a drug abuse or dependence that is neither for opioid or alcohol	

	(Other Drug Abuse and Dependence Value Set), place the member in the other drug cohort. If the member has multiple substance use diagnoses on the same claim, report the member in all SUD diagnosis stratifications for which they meet criteria.	
	Note: The total is not a sum of the diagnosis cohorts. Count members in the total denominator rate if they had at least one alcohol, opioid, or other drug abuse or dependence diagnosis during the measurement period. Report member with multiple diagnoses on the Index Episode claim only once for the total rate for the denominator.	
	**Step 3**<br>Test for Negative Diagnosis History.	
	Exclude members who had a claim/encounter with a diagnosis of AOD abuse or dependence (AOD Abuse and Dependence Value Set), Medication Assisted Treatment Value Set or a MAT dispensing event (MAT for Alcohol Abuse or Dependence Medications List; MAT for Opioid Abuse or Dependence Medications List) during the 60 days (2 months) before the IESD.	
	Note: For an inpatient IESD, use the admission date to determine the 60-day Negative Diagnosis History period. For an ED visit that results in an inpatient stay, use the ED date of service to determine the 60-day Negative Diagnosis History period. When an ED visit and an inpatient stay are billed on separate claims, the visit results in an inpatient stay when the admission date for the inpatient stay occurs on the ED date of service or one calendar day after. An ED visit billed on the same claim as an inpatient stay is considered a visit that resulted in an inpatient stay.	
	**Step 4**<br>Calculate continuous enrollment.	
	Members must be continuously enrolled for 60 days (2 months) before the IESD through 48 days after the IESD (109 total days), with no gaps.	

Index Episode	The earliest inpatient, intensive outpatient, partial hospitalization, outpatient, telehealth, detoxification, or ED visit during the Intake period with a diagnosis of SUD abuse or dependence.	
Index Episode Start Date (IESD)	The earliest date of service for an inpatient, intensive outpatient, partial hospitalization, outpatient, detoxification, or ED encounter during the Intake period with a diagnosis of SUD	
	**For an outpatient,** intensive outpatient, partial hospitalization, telehealth, detoxification, or ED visit (not resulting in an inpatient stay), the IESD is the date of service.	
	**For an inpatient** stay, the IESD is the date of discharge.	
	**For an ED visit that results in an inpatient stay**, the IESD is the date of the inpatient discharge (an SUD diagnosis is not required for the inpatient stay). When an ED visit and an inpatient stay are billed on separate claims, the visit results in an inpatient stay when the admission date for the inpatient stay occurs on the ED date of service or one calendar day after. An ED visit billed on the same claim as an inpatient stay is considered a visit that resulted in an inpatient stay.	
	**For direct transfers**, the IESD is the discharge date from the last admission (an SUD diagnosis is not required for the transfer).	
Direct transfer	A direct transfer is when the discharge date from one inpatient setting and the admission date to a second inpatient setting are one calendar day apart or less. For example:	
	• An inpatient discharge on June 1, followed by an admission to another inpatient setting on June 1, is a direct transfer.	
	• An inpatient discharge on June 1, followed by an admission to an inpatient setting on June 2, is a direct transfer.	
	• An inpatient discharge on June 1, followed by an admission to another inpatient setting on June 3, is not a direct transfer; these are two distinct inpatient stays.	

	Use the following method to identify admissions to and discharges from inpatient settings.	
	1. Identify all acute and nonacute inpatient stays (Inpatient Stay Value Set).	
	2. Identify the admission and discharge dates for the stay.	
Negative Dx History	**A period of 60 days (2 months) before the IESD when the member had no claims/encounters with a diagnosis of SUD dependence.**	
	**For an inpatient stay,** use the admission date to determine the Negative Diagnosis History.	
	**For ED visits** that result in an inpatient stay, use the ED date of service to determine the Negative Diagnosis History. An ED visit results in an inpatient stay when an ED visit and an inpatient stay are billed on separate claims. The visit results in an inpatient stay when the admission date for the inpatient stay occurs on the ED date of service or one calendar day after. An ED visit billed on the same claim as an inpatient stay is considered a visit that resulted in an inpatient stay.	
	**For direct transfers,** use the first admission to determine the Negative Diagnosis History.	
**References**		
	HEDIS measure specifications (2018) QRS Measure Technical Specifications	
	HEDIS for the Quality Rating System Value Set Directory_2016-2018	
**Claims data code:**		
	1. Identify all acute and nonacute inpatient stays (Inpatient Stay Value Set).	
	2. Identify the admission and discharge dates for the stay.	
Negative Dx History	**A period of 60 days (2 months) before the IESD when the member had no claims/encounters with a diagnosis of SUD dependence.**	
	**For an inpatient stay,** use the admission date to determine the Negative Diagnosis History.	

	**For ED visits** that result in an inpatient stay, use the ED date of service to determine the Negative Diagnosis History. An ED visit results in an inpatient stay when an ED visit and an inpatient stay are billed on separate claims. The visit results in an inpatient stay when the admission date for the inpatient stay occurs on the ED date of service or one calendar day after. An ED visit billed on the same claim as an inpatient stay is considered a visit that resulted in an inpatient stay.	
	**For direct transfers,** use the first admission to determine the Negative Diagnosis History.	
**References**		
	HEDIS Measure Specifications. QRS Measure Technical Specifications 2018. Accessed April 24, 2022. https://www.cms.gov/Medicare/Quality-Initiatives-Patient-Assessment-Instruments/QualityInitiativesGenInfo/Downloads/Revised_QRS-2018-Measure-Tech-Specs_20170929_508.pdf	
	HEDIS for the Quality Rating System Value Set Directory_2016-2018	
**Claims data code:**		
All the value sets mentioned in the algorithms are defined in the NCQA reports.	See the detailed codes in Value Set Directory	
**Indicator**	**SUD Treatment Engagement**	**Notes**
**Sample inclusion criteria**		
**Age**	18-64	
Intake period	January 1 – November 15 of the measurement year. The Intake period is used to capture new episodes.	
Continuous enrollment time	One month prior to the IESD through one month after the IESD. See the IESD definition below.<br>Build the indicator in two ways: with and without this continuous enrollment restriction.	

SUD condition	New episode of SUD during the Intake period	
**Measure**		
Rate of engagement		
**Numerator**	Numerator compliant for the Initiation of SUD Treatment numerator **AND**	
	1. Two or more inpatient admissions, outpatient visits, telehealth, intensive outpatient encounters, or partial hospitalizations with a diagnosis matching the IESD diagnosis, beginning on the day after the initiation encounter through 29 days after the initiation event (29 total days). Multiple engagement visits may occur on the same day, but they must be with different providers in order to count. Any of the following code combinations meet criteria:	
	- An acute or nonacute inpatient admission with a diagnosis matching the IESD diagnosis cohort using one of the following: Alcohol Abuse and Dependence Value Set, Opioid Abuse and Dependence Value Set, Other Drug Abuse and Dependence Value Set. To identify acute or nonacute inpatient admissions:	
	1. Identify all acute and nonacute inpatient stays (Inpatient Stay Value Set).	
	2. Identify the admission date for the stay.	
	- IET Stand Alone Visits Value Set with a diagnosis matching the IESD diagnosis cohort using one of the following: Alcohol Abuse and Dependence Value Set, Opioid Abuse and Dependence Value Set, Other Drug Abuse and Dependence Value Set, with or without a telehealth modifier (Telehealth Modifier Value Set).	
	- IET Visits Group 1 Value Set with IET POS Group 1 Value Set with a diagnosis matching the IESD diagnosis cohort using one of the following: Alcohol Abuse and Dependence Value Set, Opioid Abuse and Dependence Value Set, Other Drug Abuse and Dependence Value Set, with or without a telehealth modifier (Telehealth Modifier Value Set).	
	- IET Visits Group 2 Value Set with IET POS Group 2 Value Set with a diagnosis matching the IESD diagnosis cohort using one of the following: Alcohol Abuse and Dependence Value Set, Opioid Abuse and Dependence Value Set, Other	

	Drug Abuse and Dependence Value Set, with or without a telehealth modifier (Telehealth Modifier Value Set).	
	- A telephone visit (Telephone Visits Value Set) with a diagnosis matching the IESD diagnosis cohort using one of the following: Alcohol Abuse and Dependence Value Set, Opioid Abuse and Dependence Value Set, Other Drug Abuse and Dependence Value Set.	
	- An online assessment (Online Assessments Value Set) with a diagnosis matching the IESD diagnosis cohort using one of the following: Alcohol Abuse and Dependence Value Set, Opioid Abuse and Dependence Value Set, Other Drug Abuse and Dependence Value Set.	
	2. If the Initiation of SUD treatment was not a MAT dispensing event, one or more of the MAT dispensing events (MAT for Alcohol Abuse or Dependence Medications List; MAT for Opioid Abuse or Dependence Medications List) beginning on the day after the initiation encounter through 33 days after the initiation event (total of 34 days).	
	- If the Initiation of SUD treatment was for treatment ofr a diagnosis of alcohol abuse or dependence (Alcohol Abuse and Dependence Value Set), one or more MAT dispensing events (MAT for Alcohol Abuse or Dependence Medications List) or claims for MAT (Medication Assisted Treatment Value Set), beginning on the day after the initiation encounter through 33 days after the initiation event (total of 34 days), meets criteria for Alcohol Abuse and Dependence Treatment.	
	- If the Initiation of SUD treatment was for treatment of for a diagnosis of opioid abuse or dependence (Opioid Abuse and Dependence Value Set), one or more MAT dispensing events (MAT for Opioid Abuse or Dependence Medications List) or claims for MAT (Medication Assisted Treatment Value Set), beginning on the day after the initiation encounter through 33 days after the initiation event (total of 34 days), meets criteria for Opioid Abuse and Dependence Treatment.	

	3. If the Initiation of SUD treatment was a MAT dispensing event, two or more engagement events where at least one meets criteria for 1. For example, two engagement events from criteria 2 do not meet numerator compliance.	
	Note: If the member is compliant for multiple cohorts, only count the member once for the Total Engagement numerator. The Total Column is not the sum of the diagnosis columns.<br><br>For members who initiated treatment via an inpatient admission, the 33-day **period for the two engagement visits begins** the day after discharge.<br><br>The time frame for engagement, which includes the initiation event, is 34 total days.	
**Denominator**	Same denominator as for *SUD treatment Initiation* algorithm, except without the continuous enrollment requirement.	
**References**		
	HEDIS Measure Specifications. QRS Measure Technical Specifications 2018. Accessed April 24, 2022. https://www.cms.gov/Medicare/Quality-Initiatives-Patient-Assessment-Instruments/QualityInitiativesGenInfo/Downloads/Revised_QRS-2018-Measure-Tech-Specs_20170929_508.pdf	
	HEDIS for the Quality Rating System Value Set Directory_2016-2018	
**Claims data code:**		
	HEDIS for the Quality Rating System Value Set Directory_2016-2018	See the detailed codes in Value Set Directory
All the value sets mentioned in the algorithms are defined in the NCQA reports.	See the detailed codes in Value Set Directory	
**Indicator**	**Engagement: Alcohol Pharmacotherapy**	**Notes**
**Sample inclusion criteria**		

Age	18-64	
Intake period	January 1–November 15 of the measurement year. The Intake period is used to capture new episodes.	
Continuous enrollment time	One month prior to the IESD through one month after the IESD. See the IESD definition below.<br>Build the indicator in two ways: with and without this continuous enrollment restriction.	
SUD condition	New episode of SUD during the Intake period	
**Measure**		
Rate of engagement	% of initiation after the index visit	
Numerator	Number of individuals who initiate pharmacotherapy with at least one prescription for an alcohol treatment medication within 30 days following index visit with a diagnosis of alcohol abuse	
Denominator	Number of individuals with index visit associated with an alcohol abuse diagnosis after 60-day clean period with no SUD claims (negative Dx history)	
How to operationalize	Create a binary indicator which equals "1" if there was at least one prescription for AUD within 30 days of Dx of AUD (at visit/encounter level). Then calculate the mean or % of 1s.	
**References**		
	Thomas CP, Ritter GA, Harris AHS, Garnick DW, Freedman KI, Herbert B. Applying American Society of Addiction Medicine Performance Measures in Commercial Health Insurance and Services Data. J Addict Med. 2018;12(4):287-294.	Use for AUD Medication Treatment
	Harris AH, Weisner CM, Chalk M, Capoccia V, Chen C, Thomas CP. Specifying and Pilot Testing Quality Measures for the American Society of Addiction Medicine's Standards of Care. J Addict Med. 2016;10(3):148-155.	Use for AUD Medication Treatment
	Mattke S, Predmore Z, Sloss E, Wilks A, Watkins KE. Evidence for Misspecification of a Nationally Used Quality Measure for Substance Use Treatment. J Healthc Qual. 2018;40(4):228-235.	Use for AUD Medication Treatment

	HEDIS Measure Specifications. QRS Measure Technical Specifications 2018. Accessed April 24, 2022. https://www.cms.gov/Medicare/Quality-Initiatives-Patient-Assessment-Instruments/QualityInitiativesGenInfo/Downloads/Revised_QRS-2018-Measure-Tech-Specs_20170929_508.pdf	Use for defining index visit and negative Dx history
	HEDIS for the Quality Rating System Value Set Directory_2016-2018	Use for defining index visit and negative Dx history
**Claims data code:**		
Alcohol use disorder (AUD)	Search the primary and secondary diagnosis for the codes listed in Alcohol Abuse	Source: Thomas et al. (2018)
Prescription for AUD	See "Med for AUD", "Med for Nicotine' and "Med for Cocaine" in the NDC directory<br>Include:	Source: Harris et al. (2016), Thomas et al. (2018) for OUD medications. Mattke et al. (2018), Thomas et al. (2018): on top of the list of Harris, adds methadone. For AUD medications, source: Harris et al. (2016): use disorder treatment, which is not FDA-approved but guideline-recommended based on strong evidence from a meta-analysis. Mattke et al. (2018) and Thomas et al. (2018).

	Injectable naltrexone was identified based on a HCPCS code (J2315) in the medical claims. Buprenorphine service or implant can be identified with HCPCS codes J0570, J0571, J0572, J0573, J0574, J0575, and J0592. As methadone may only be used for MAT through in-office dispensing in licensed treatment centers, the methadone (OTP) service was also identified based on a HCPCS code (H0020 and S0109).	Source: Harris et al. (2016), Thomas et al. (2018)
Index Episode	The earliest inpatient, intensive outpatient, partial hospitalization, outpatient, telehealth, detoxification, or ED visit during the Intake period with a diagnosis of SUD abuse or dependence (Comprehensive SUD Dx Value Set)	Source: HEDIS measure specifications (2018) QRS Measure Technical Specifications
Index Episode Start Date (IESD)	The earliest date of service for an inpatient, intensive outpatient, partial hospitalization, outpatient, detoxification, or ED encounter during the Intake Period with a diagnosis of SUD	Source: HEDIS measure specifications (2018) QRS Measure Technical Specifications
	**For an outpatient,** intensive outpatient, partial hospitalization, telehealth, detoxification, or ED visit (not resulting in an inpatient stay), the IESD is the date of service.	
	**For an inpatient** stay, the IESD is the date of discharge.	
	For an ED visit that results in an inpatient stay, the IESD is the date of the inpatient discharge (an SUD diagnosis is not required for the inpatient stay). When an ED visit and an inpatient stay are billed on separate claims, the visit results in an inpatient stay when the admission date for the inpatient stay occurs on the ED date of service or one calendar day after. An ED visit billed on the same claim as an inpatient stay is considered a visit that resulted in an inpatient stay.	
	For direct transfers, the IESD is the discharge date from the last admission (an SUD diagnosis is not required for the transfer).	

Direct transfer	A direct transfer is when the discharge date from one inpatient setting and the admission date to a second inpatient setting are one calendar day apart or less. For example:	Source: HEDIS measure specifications (2018) QRS Measure Technical Specifications
	•An inpatient discharge on June 1, followed by an admission to another inpatient setting on June 1, is a direct transfer.	Source: HEDIS for the Quality Rating System Value Set Directory_2016-2018
	•An inpatient discharge on June 1, followed by an admission to an inpatient setting on June 2, is a direct transfer.	
	•An inpatient discharge on June 1, followed by an admission to another inpatient setting on June 3, is not a direct transfer; these are two distinct inpatient stays.	
	Use the following method to identify admissions to and discharges from inpatient settings.	
	1. Identify all acute and nonacute inpatient stays (Inpatient Stay Value Set).	
	2. Identify the admission and discharge dates for the stay.	
Index Visit	Identified as the visit occurred on the index episode start date	Source: HEDIS measure specifications (2018) QRS Measure Technical Specifications
Negative Dx History	**A period of 60 days (2 months) before the IESD when the member had no claims/ encounters with a diagnosis of SUD dependence (Comprehensive SUD Dx Value Set).**	Source: HEDIS measure specifications (2018) QRS Measure Technical Specifications
	**For an inpatient stay,** use the admission date to determine the Negative Diagnosis History.	

	**For ED visits** that result in an inpatient stay, use the ED date of service to determine the Negative Diagnosis History. An ED visit results in an inpatient stay when an ED visit and an inpatient stay are billed on separate claims, the visit results in an inpatient stay when the admission date for the inpatient stay occurs on the ED date of service or one calendar day after. An ED visit billed on the same claim as an inpatient stay is considered a visit that resulted in an inpatient stay.	
	**For direct transfers,** use the first admission to determine the Negative Diagnosis History.	
**Indicator**	**Engagement: Opioid Pharmacotherapy**	**Notes**
**Sample inclusion criteria**		
Age	18-64	
Intake period	January 1 – November 15 of the measurement year. The Intake period is used to capture new episodes.	
Continuous enrollment time	One month prior to the IESD through one month after the IESD. See the IESD definition below.<br>Build the indicator in two ways: with and without this continuous enrollment restriction.	
SUD condition	New episode of SUD during the Intake period	
**Measure**		
Rate of engagement	% of initiation after the index visit	
Numerator	Number of individuals who initiate pharmacotherapy with at least one prescription for an opioid treatment medication within 30 days following index visit with a diagnosis of opioid dependence abuse	
Denominator	Number of individuals with index visit associated with an opioid dependence abuse diagnosis after 60-day clean period with no SUD claims (negative Dx history)	

How to operationalize	Create a binary indicator which equals "1" if there was at least one prescription for OUD within 30 days of Dx of OUD (at visit/encounter level). Then calculate the mean or % of 1s.	
**References**		
	Thomas CP, Ritter GA, Harris AHS, Garnick DW, Freedman KI, Herbert B. Applying American Society of Addiction Medicine Performance Measures in Commercial Health Insurance and Services Data. J Addict Med. 2018;12(4):287-294.	Use for OUD Medication Treatment
	Harris AH, Weisner CM, Chalk M, Capoccia V, Chen C, Thomas CP. Specifying and Pilot Testing Quality Measures for the American Society of Addiction Medicine's Standards of Care. J Addict Med. 2016;10(3):148-155.	Use for OUD Medication Treatment
	Mattke S, Predmore Z, Sloss E, Wilks A, Watkins KE. Evidence for Misspecification of a Nationally Used Quality Measure for Substance Use Treatment. J Healthc Qual. 2018;40(4):228-235.	Use for OUD Medication Treatment
	HEDIS Measure Specifications. QRS Measure Technical Specifications 2018. Accessed April 24, 2022. https://www.cms.gov/Medicare/Quality-Initiatives-Patient-Assessment-Instruments/QualityInitiativesGenInfo/Downloads/Revised_QRS-2018-Measure-Tech-Specs_20170929_508.pdf	Use for defining index visit and negative Dx history
	HEDIS for the Quality Rating System Value Set Directory_2016-2018	Use for defining index visit and negative Dx history
**Claims data code:**		
Opioid use disorder (OUD)	Search the primary and secondary diagnosis for the codes listed in Opioid Abuse	Source: Thomas et al. (2018)

Prescription for AUD and OUD	See "Med for OUD", "Med for Nicotine" and "Med for Cocaine" in the NDC directory<br>Include:	Source: Harris et al. (2016), Thomas et al. (2018) for OUD medications. Mattke et al. (2018), Thomas et al. (2018): on top of the list of Harris, adds methadone. For AUD medications, source: Harris et al. (2016): use disorder treatment, which is not FDA-approved but guideline-recommended based on strong evidence from a meta-analysis. Mattke et al. (2018) and Thomas et al. (2018).
	Injectable naltrexone was identified based on a HCPCS code (J2315) in the medical claims. Buprenorphine service or implant can be identified with HCPCS codes J0570, J0571, J0572, J0573, J0574, J0575, and J0592. As methadone may only be used for MAT through in-office dispensing in licensed treatment centers, the methadone (OTP) service was also identified based on a HCPCS code (H0020 and S0109).	Source: Harris et al. (2016), Thomas et al. (2018)
Index Episode	The earliest inpatient, intensive outpatient, partial hospitalization, outpatient, telehealth, detoxification, or ED visit during the Intake Period with a diagnosis of SUD abuse or dependence (Comprehensive SUD Dx Value Set)	Source: HEDIS measure specifications (2018) QRS Measure Technical Specifications

Index Episode Start Date (IESD)	The earliest date of service for an inpatient, intensive outpatient, partial hospitalization, outpatient, detoxification, or ED encounter during the Intake Period with a diagnosis of SUD	Source: HEDIS measure specifications (2018) QRS Measure Technical Specifications
	**For an outpatient,** intensive outpatient, partial hospitalization, telehealth, detoxification, or ED visit (not resulting in an inpatient stay), the IESD is the date of service.	
	**For an inpatient** stay, the IESD is the date of discharge.	
	For an ED visit that results in an inpatient stay, the IESD is the date of the inpatient discharge (an SUD diagnosis is not required for the inpatient stay). When an ED visit and an inpatient stay are billed on separate claims, the visit results in an inpatient stay when the admission date for the inpatient stay occurs on the ED date of service or one calendar day after. An ED visit billed on the same claim as an inpatient stay is considered a visit that resulted in an inpatient stay.	
	For direct transfers, the IESD is the discharge date from the last admission (an SUD diagnosis is not required for the transfer).	
Direct transfer	A direct transfer is when the discharge date from one inpatient setting and the admission date to a second inpatient setting are one calendar day apart or less. For example:	Source: HEDIS measure specifications (2018) QRS Measure Technical Specifications
	•An inpatient discharge on June 1, followed by an admission to another inpatient setting on June 1, is a direct transfer.	Source: HEDIS for the Quality Rating System Value Set Directory_2016-2018
	•An inpatient discharge on June 1, followed by an admission to an inpatient setting on June 2, is a direct transfer.	
	•An inpatient discharge on June 1, followed by an admission to another inpatient setting on June 3, is not a direct transfer; these are two distinct inpatient stays.	

	Use the following method to identify admissions to and discharges from inpatient settings.	
	1. Identify all acute and nonacute inpatient stays (Inpatient Stay Value Set).	
	2. Identify the admission and discharge dates for the stay.	
Index Visit	Identified as the visit occurred on the index episode start date	Source: HEDIS measure specifications (2018) QRS Measure Technical Specifications
Negative Dx History	**A period of 60 days (2 months) before the IESD when the member had no claims/ encounters with a diagnosis of SUD dependence (Comprehensive SUD Dx Value Set).**	Source: HEDIS measure specifications (2018) QRS Measure Technical Specifications
	**For an inpatient stay,** use the admission date to determine the Negative Diagnosis History.	
	**For ED visits** that result in an inpatient stay, use the ED date of service to determine the Negative Diagnosis History. An ED visit results in an inpatient stay when an ED visit and an inpatient stay are billed on separate claims, the visit results in an inpatient stay when the admission date for the inpatient stay occurs on the ED date of service or one calendar day after. An ED visit billed on the same claim as an inpatient stay is considered a visit that resulted in an inpatient stay.	
	**For direct transfers,** use the first admission to determine the Negative Diagnosis History.	
**Indicator**	**Smoking Cessation Engagement: Prescription/Consult; Treatment**	**Notes**
**Sample inclusion criteria**		
Age	18-64	
Enrollment time	Enrolled at least one month in a given calendar year	
SUD condition	Had a smoking related diagnosis at any time in the first six months of their eligible enrollment episode.	

Measure		
Prevalence of smoking cessation		
Numerator	Members in the denominator who also used smoking cessation services in the first six months of their eligible enrollment episode.	
Denominator	Members identified as smokers.	
Note	This algorithm comes in two versions – with bupropion and without bupropion.	
Average number of services utilized per smoker		
Numerator	The total count of smoking cessation services utilized in the first six months of members' eligible enrollment episodes.	
Denominator	Members identified as smokers.	
Note	This algorithm comes in two versions – with bupropion and without bupropion.	
**References**		
	Malloy K, Proj A, Battles H, et al. Smoking Cessation Benefit Utilization: Comparing Methodologies for Measurement using New York State's Medicaid Data. Nicotine Tob Res. 2018;20(12):1467-1473.	
	Utilization of Smoking Cessation Benefits in Medicaid Managed Care 2012-2016	
**Claims data code:**		
Identification of smokers based on diagnosis, Smoking Cessation Counseling	See value set "Smoking Cessation Treatment"	Malloy et al. (2018)
Identification Smoking Cessation pharmacotherapy	National Drug Codes (NDC) are used to identify pharmacotherapy. The identification of applicable NDC codes is based on drug therapeutic codes 72142, 72143, 72144, 72145, 72147, 72148, and 72149, or categorization of the drug as being a "smoking deterrent." Bupropion, due to its use as both a smoking cessation agent and an antidepressant is excluded from analyses, except for cases in which the drug's therapeutic code explicitly indicates that its use was for smoking cessation. Any product with a therapeutic class code of U6W (Bulk	Malloy et al. (2018)

	Chemicals) is also excluded, as these are not used in the clinical setting. In total, there are 1387 NDC codes identified and utilized in analysis.	
	Notes: The drug therapeutic codes are a code that determines the therapeutic class of the drug, and it is usually included in the claims data (e.g., MarketScan)	
**Indicator**	**SUD Tx Engagement: Follow-up w/in 30 Days After ED Visit**	**Notes**
**Sample inclusion criteria**		
Age	18-64	
SUD condition	An ED visit (ED Value Set) with a principal diagnosis of AOD abuse or dependence (AOD Abuse and Dependence Value Set) on or between January 1 and December 1 of the measurement year where the beneficiary was age 18 or older on the date of the visit.	
Exclusions	Exclude ED visits followed by an admission to an acute or nonacute inpatient care setting on the date of the ED visit or within the 30 days after the ED visit (31 total days), regardless of principal diagnosis for the admission. To identify admissions to an acute or nonacute inpatient care setting:	
	1. Identify all acute and nonacute inpatient stays (Inpatient Stay Value Set).	
	2. Identify the admission date for the stay.	
	An ED or observation visit billed on the same claim as an inpatient stay is considered a visit that resulted in an inpatient stay. These events are excluded from the measure because admission to an acute or nonacute inpatient setting may prevent an outpatient follow-up visit from taking place.	
Multiple ED visits in a 31-day period	If a beneficiary has more than one ED visit in a 31-day period, include only the first eligible ED visit. For example, if a beneficiary has an ED visit on January 1, then include the January 1 visit and do not include ED visits that occur on or between January 2 and January 31; then, if applicable, include the next ED visit	Note: Removal of multiple visits in a 31-day period is based on eligible visits. Assess each ED visit for exclusion before

	that occurs on or after February 1. Identify visits chronologically including only one per 31-day period.	removing multiple visits in a 31-day period.
Notes	Data are at the person-calendar year level. Measure is cumulative	
	This algorithm comes in two versions – "follow up occurs within 30 days" and "follow up occurs within 7 days"	
**Measure**		
Numerator for 30-day follow-up	Number of outpatient visit, intensive outpatient encounter or partial hospitalization, with any practitioner, with a primary diagnosis of SUD within 30 days after the ED visit. Include outpatient visits, intensive outpatient visits or partial hospitalizations that occur on the date of the ED visit.	
Numerator for 7-day follow-up	Number of outpatient visit, intensive outpatient encounter or partial hospitalization, with any practitioner, with a primary diagnosis of SUD within 7 days after the ED visit. Include outpatient visits, intensive outpatient visits or partial hospitalizations that occur on the date of the ED visit.	
Denominator	Members who had an "eligible ED visit" claim in the first six months of their eligible enrollment episode.	
How to operationalize	Create a binary indicator for each eligible ED visit whether there is a follow-up visit as defined in the numerator. Then calculate the mean or % of 1s.	
Eligible ED visit	• SUD condition: An ED visit with a principal diagnosis of AOD abuse or dependence.<br>• Exclusions: Exclude ED visits followed by an admission to an acute or non-acute inpatient care setting on the date of the ED visit or within the 30 days after the ED visit (31 total days), regardless of principal diagnosis for the admission.<br>• Multiple ED visits in a 31 day period: If a member has more than one ED visit in a 31-day period, include only the first eligible ED visit. For example, if a member has an ED visit on January 1, then include the January 1 visit and do not include ED visits that occur on or between January 2 and January 31; then, if applicable,	

	include the next ED visit that occurs on or after February 1. Identify visits chronologically including only one per 31-day period.	
Eligible follow-up visit	• For both indicators (7 days and 30 days), any of the following meet criteria for a follow-up visit:<br>o IET Stand Alone Visits Value Set with a principal diagnosis of AOD abuse or dependence<br>o IET Visits Group 1 Value Set with IET POS Group 1 Value Set and a principal diagnosis of AOD abuse or dependence<br>o IET Visits Group 2 Value Set with IET POS Group 2 Value Set and a principal diagnosis of AOD abuse or dependence<br>o A telephone visit with a principal diagnosis of AOD abuse or dependence<br>o An online assessment with a principal diagnosis of AOD abuse or dependence	
**References**		
	Adult Core Set Reporting Resources | Medicaid. 2018. https://www.medicaid.gov/medicaid/quality-of-care/performance-measurement/adult-and-child-health-care-quality-measures/adult-core-set-reporting-resources/index.html Accessed April 24, 2022.	Definition of indicator
	National Committee for Quality Assurance. (2016). Propose new measures for hedis 2017: Follow-up after emergency department visit for mental illness and alcohol and other drug dependence. Accessible at: https://www.ncqa.org/hedis/measures/follow-up-after-emergency-department-visit-for-alcohol-and-other-drug-abuse-or-dependence/	
	HEDIS for the Quality Rating System Value Set Directory_2016-2018	
**Claims data code:**		

Identification of follow-up visit	For both indicators (7 days and 30 days), any of the following meet criteria for a follow-up visit:	CMS uses the same measure in their Core Set of Health Care Quality Measures for Medicaid Adult Enrollees (2018). Originally proposed in National Committee for Quality Assurance. (2016). Proposed in new measures for HEDIS 2017: Follow-up after emergency department visit for mental illness and alcohol and other drug dependence.
	IET Stand Alone Visits Value Set with a principal diagnosis of AOD abuse or dependence (AOD Abuse and Dependence Value Set), with or without a telehealth modifier (Telehealth Modifier Value Set)	Source: HEDIS for the Quality Rating System Value Set Directory_2016-2018
	IET Visits Group 1 Value Set with IET POS Group 1 Value Set and a principal diagnosis of AOD abuse or dependence (AOD Abuse and Dependence Value Set), with or without a telehealth modifier (Telehealth Modifier Value Set)	
	IET Visits Group 2 Value Set with IET POS Group 2 Value Set and a principal diagnosis of AOD abuse or dependence (AOD Abuse and Dependence Value Set), with or without a telehealth modifier (Telehealth Modifier Value Set)	
	A telephone visit (Telephone Visits Value Set) with a principal diagnosis of AOD abuse or dependence (AOD Abuse and Dependence Value Set)	
	An online assessment (Online Assessments Value Set) with a principal diagnosis of AOD abuse or dependence (AOD Abuse and Dependence Value Set)	

Indicator	Identification of Comorbidity with Mental Illness	Notes

**Sample inclusion criteria**		
Age	18-64	
Enrollment time	Enrolled at least one month in a given calendar year	
SUD condition	Had a SUD diagnosis/services at anytime in the enrolled year	
Notes	Based on Clark et al. 2007, the comorbidity was identified solely through ICD-9. Having MH treatment (CPT codes) but no MH Dx is not considered as a way of identifying MH illness.	
**Measure**		
Numerator	# of enrollees who also had mental health illness among those included in the denominator	
Denominator	# of eligible enrollees who were identified with SUD in the first six months of their eligible enrollment episode.	
How to operationalize	First, create a binary indicator, which equals "1" if an enrollee was identified as having an SUD (in outpatient, inpatient, or ED claims) and at least 1 MH Dx in that given year and "0" otherwise. Based on the individual-level indicator, it is straightforward to calculate the proportion of enrollees with SUD Dx for a given plan in a given year.	
**References**		
	Clark RE, Samnaliev M, McGovern MP. Treatment for co-occurring mental and substance use disorders in five state Medicaid programs. Psychiatr Serv. 2007;58(7):942-948.	
	Clark RE, Samnaliev M, McGovern MP. Impact of substance disorders on medical expenditures for medicaid beneficiaries with behavioral health disorders. Psychiatr Serv. 2009;60(1):35-42.	
**Claims data code:**		
Identification of Mental illness through MH Dx	See value set "Mental Illness"	Source: Clark, Samnaliev, McGovern (2009)

Identification of SUD diagnosis/services	Includes presence of either a SUD Dx, prescription of medication commonly used for addiction treatment, or procedures specifically for alcohol or drug disorder treatment. Use the same identification strategy as for "Presence of a Substance Use Disorders (SUD)". See details below:	
ICD-9/10 codes for SUD Dx	See value set "Comprehensive SUD Dx"	
Medications		
Prescription	See "Med for OUD", "Med for AUD", "Med for Nicotine" and "Med for Cocaine" in the NDC directory	
Injectable or Service	Injectable naltrexone was identified based on HCPCS code (J2315) in the medical claims. Buprenorphine service or implant can be identified with HCPCS codes J0570, J0571, J0572, J0573, J0574, J0575, and J0592. As methadone may only be used for MAT through in-office dispensing in licensed treatment centers, the methadone (OTP) service was also identified based on a HCPCS code (H0020 and S0109).	
Detoxification or rehabilitation procedures specifically for alcohol or drug disorder treatment	See value set "Detoxification"	
**Indicator**	**Monthly expenditures on SUD Tx (Mean, including zeros)**	**Notes**
**Sample inclusion criteria**		
Age	18-64	
Enrollment time	Enrolled in current month	
SUD condition	Treated for SUD in the current month	
	SUD treatment defined as either an ICD-9 code	
**Measure**		
Count	Number of people treated for SUD that month	
Amount	Total paid per month	
SUD expenditure - Mean	Mean of a member's average monthly expenditures, for SUD treatment, including zeros.	

**References**		
	Clark RE, Baxter JD, Aweh G, O'Connell E, Fisher WH, Barton BA. Risk Factors for Relapse and Higher Costs Among Medicaid Members with Opioid Dependence or Abuse: Opioid Agonists, Comorbidities, and Treatment History. J Subst Abuse Treat. 2015;57:75-80.	
	Clark RE, Samnaliev M, McGovern MP. Impact of substance disorders on medical expenditures for medicaid beneficiaries with behavioral health disorders. Psychiatr Serv. 2009;60(1):35-42.	Expenditure definitions
**Claims data coding**		
SUD Treatment (Tx)	Inpatient, intensive outpatient, partial hospitalization, outpatient, telehealth, detoxification, or ED visit with a diagnosis of SUD abuse or dependence (SUD Dx), or filled prescription for medications commonly used to treat addiction	Source: HEDIS measure specifications (2018) QRS Measure Technical Specifications. Detoxification is not considered to be SUD treatment
ICD-9/10 codes for SUD Dx	See value set "Comprehensive SUD Dx"	
Medications		
Prescription	See "Med for OUD", "Med for AUD", "Med for Nicotine" and "Med for Cocaine" in the NDC directory	
Injectable or Service	Injectable naltrexone was identified based on a HCPCS code (J2315) in the medical claims. Buprenorphine service or implant can be identified with HCPCS codes J0570, J0571, J0572, J0573, J0574, and J0575. As methadone may only be used for MAT through in-office dispensing in licensed treatment centers, the methadone (OTP) service was also identified based on a HCPCS code (H0020 and S0109).	
Note	Source of the code is included in the value set document.	
**Indicator**	**Percentage of non-zero expenditures on SUD Tx**	**Notes**
**Sample inclusion criteria**		

Age	18-64	
Enrollment time	Enrolled in current month	
SUD condition	Treated for SUD in the current month	
	SUD treatment defined as either an ICD-9 code	
**Measure**		
Count	Number of people treated for SUD that month	
Amount	Total paid per month	
SUD expenditure - Percentage	Percentage of a member's monthly expenditure on SUD treatment from total amount paid per month, excluding zeros.	
**References**		
	Clark RE, Baxter JD, Aweh G, O'Connell E, Fisher WH, Barton BA. Risk Factors for Relapse and Higher Costs Among Medicaid Members with Opioid Dependence or Abuse: Opioid Agonists, Comorbidities, and Treatment History. J Subst Abuse Treat. 2015;57:75-80.	
	Clark RE, Samnaliev M, McGovern MP. Impact of substance disorders on medical expenditures for medicaid beneficiaries with behavioral health disorders. Psychiatr Serv. 2009;60(1):35-42.	Expenditure definitions
**Claims data coding**		
SUD Treatment (Tx)	Inpatient, intensive outpatient, partial hospitalization, outpatient, telehealth, detoxification, or ED visit with a diagnosis of SUD abuse or dependence (SUD Dx), or filled prescription for medications commonly used to treat addiction	Source: HEDIS measure specifications (2018) QRS Measure Technical Specifications. Detoxification is not considered to be SUD treatment
ICD-9/10 codes for SUD Dx	See value set "Comprehensive SUD Dx"	
Prescription	See "Med for OUD", "Med for AUD", "Med for Nicotine" and "Med for Cocaine" in the NDC directory	

Injectable or Service	Injectable naltrexone was identified based on a HCPCS code (J2315) in the medical claims. Buprenorphine service or implant can be identified with HCPCS codes J0570, J0571, J0572, J0573, J0574, and J0575.As methadone may only be used for MAT through in-office dispensing in licensed treatment centers, the methadone (OTP) service was also identified based on a HCPCS code (H0020 and S0109).	
Note	Source of the code is included in the value set document.	
**Indicator**	**Receipt of Psychosocial interventions**	**Notes**
**Sample inclusion criteria**		
Age	18-64	
Enrollment time	Enrolled at least one month in a given calendar year	
SUD condition	Had SUD in a given year	
**Measure**		
Numerator	Number of eligible population with SUD and received at least one psychosocial interventions in the first six months of their eligible enrollment episode.	
Denominator	Number of adult enrollees with SUD in the first six months of their eligible enrollment episode.	
**References**		
	Finnerty M, Neese-Todd S, Pritam R, et al. Access to Psychosocial Services Prior to Starting Antipsychotic Treatment Among Medicaid-Insured Youth. J Am Acad Child Adolesc Psychiatry. 2016;55(1):69-76.e3.	Includes CPT codes
	McHugh RK, Hearon BA, Otto MW. Cognitive behavioral therapy for substance use disorders. Psychiatr Clin North Am. 2010;33(3):511-525.	Details efficacy and modes of CBT for SUD Disorders (individual and group treatments: motivational interventions, contingency

		management, relapse prevention); couples and family treatment; combination treatment strategies. Does not touch on insurance claims/reimbursements.
	Clark RE, Teague GB, Ricketts SK, et al. Cost-effectiveness of assertive community treatment versus standard case management for persons with co-occurring severe mental illness and substance use disorders. Health Serv Res.1998;33(5):1285-1308.	Cost effectiveness analysis between standard case management programs vs. assertive community treatment teams among people with co-occurring SUD and mental illness. They measured consumption of services (general healthcare costs, legal system costs, other community service costs) using Medicaid data, among other sources. No specific information on claim codes available.

	Drake RE, O'Neal EL, Wallach MA. A systematic review of psychosocial research on psychosocial interventions for people with co-occurring severe mental and substance use disorders. J Subst Abuse Treat. 2008;34(1):123-138.	Review article: interventions for adults w co-occurring mental health illness and substance use disorders (US+international): individual counseling, group counseling (6+ months), family intervention, case management (6 mo - 3 yrs), residential treatment (6mo - 1+yr), intensive outpatient rehab, contingency management, and legal intervention (e.g., jail diversion, release programs).
	Terplan M, Ramanadhan S, Locke A, Longinaker N, Lui S. Psychosocial interventions for pregnant women in outpatient illicit drug treatment programs compared to other interventions. Cochrane Database Syst Rev. 2015;(4):CD006037.	Review article on SUD psychosocial interventions in pregnancy: contingency management (e.g., vouchers, other incentives) and motivational interviewing approaches.
**Claims data code:**		

Identification of psychosocial interventions	See Value Set "Psychosocial Interventions"	

Outcomes

Indicator	Rapid Readmission to Inpatient SUD Care 2-30 days Post-Discharge	Notes
**Sample inclusion criteria**		
Age	18-64	
Enrollment time	Enrolled at least one month in a given calendar year	
SUD condition	Had acute SUD treatment at any time in the first six months of their eligible enrollment episode.	
Notes	This algorithm comes in two versions – readmission within 2-30 day	
**Measure**		
Numerator	Number of adults in the denominator who had a readmission to the same level of care within either 2 to 30 days	
Denominator	Adults with acute SUD admission in the first six months of their eligible enrollment episode.	
**References**		
	American Society of Addiction Medicine. The ASAM Performance Measures. Chevy Chase, MD: American Society of Addiction Medicine (ASAM);2014.	Referenced for SUD determination
	Daley MC. Race, managed care, and the quality of substance abuse treatment. Adm Policy Ment Health. 2005;32(4):457-476.	Source of the 7-30 day determination of rapid re-admission.
	Shepard DS et al., “Managed Care and the Quality of Substance Abuse Treatment,” Journal of Mental Health Policy and Economics. 2002; 5(4):163 –174.	Defines episode and readmission
	Korkeila, JA et al, Frequently hospitalized psychiatric patients: A study of predictive factors. Social Psychiatry and Psychiatric Epidemiology.1998;33:528-534.	Suggest 8 days between initial claim and re-admission.
	Clark RE, Baxter JD, Aweh G, O'Connell E, Fisher WH, Barton BA. Risk Factors for Relapse and Higher Costs Among Medicaid Members with Opioid Dependence or Abuse: Opioid Agonists, Comorbidities, and Treatment History. J Subst Abuse Treat. 2015;57:75-80.	Defines relapse measures
**Claims data code:**		

ICD-9/10 codes for SUD Dx	See value set "Comprehensive SUD Dx"	
Codes for acute SUD treatment (inpatient, ED, and/or Detox)	See Value Set for the sheet "Detoxification"	Shepard et al. (2002) Clark et al. (2015)
	See Value Set for the sheet "ED" **with an SUD diagnosis (Clark et al. 2015 requires a primary Dx of SUD for defining the relapse measure)** or	
	See Value Set for the sheet "Inpatient Stay" with a SUD diagnosis **(Clark et al. 2015 requires a primary Dx of SUD for defining the relapse measure)**	
Definition of Episode	For inpatient and residential treatment, an episode is defined as any sequential claims of the same modality with 0 or 1 days between the discharge date of the first claim and the admission date of the second claims.	Shepard et al. (2002)
Definition of Rapid Re-admission	For inpatient and residential treatment, an admission to the same level (meaning in ED, inpatient or detoxification) of care within 2 to 30 days, is considered as 30-day rapid re-admission.	Shepard et al. (2002)
	For the 7-day rapid-readmission, an admission to the same level of care within 2 to 7 days is considered as rapid re-admission.	Shepard et al. (2002)
**Indicator**	**Relapse**	**Notes**
**Sample inclusion criteria**		
Age	18-64	
Enrollment time	Enrolled at least one month in a given calendar year	
SUD condition	Had SUD in the first six months of their eligible enrollment episode.	
Notes	This algorithm comes in two versions – relapse within 2-30 days of the original claim; and relapse within 7-30 days of the original claim.	
**Measure**		
Numerator	Number of eligible populations who had least one relapse event within 2 – 30 days of the original claim.	
Denominator	Members who had an SUD treatment in the first six months of their eligible enrollment episode.	

Notes	Create binary indicator equals one if a member received any relapse treatment based on the codes provided below and 0 otherwise.	
**References**		
	Clark RE, Baxter JD, Aweh G, O'Connell E, Fisher WH, Barton BA. Risk Factors for Relapse and Higher Costs Among Medicaid Members with Opioid Dependence or Abuse: Opioid Agonists, Comorbidities, and Treatment History. J Subst Abuse Treat. 2015;57:75-80.	
	Zhang Y, Kwong TC. Utilization management in toxicology. Clin Chim Acta. 2014;427:158-166.	
	Clark RE, Samnaliev M, Baxter JD, Leung GY. The evidence doesn't justify steps by state Medicaid programs to restrict opioid addiction treatment with buprenorphine. Health Aff (Millwood). 2011;30(8):1425-1433.	Description of relapse measures
	Shepard DS et al., “Managed Care and the Quality of Substance Abuse Treatment,” Journal of Mental Health Policy and Economics. 2002; 5(4):163 –174.	Defines Episode and readmission
**Claims data code:**		
SUD Treatment (Tx)	Inpatient, intensive outpatient, partial hospitalization, outpatient, telehealth, detoxification, or ED visit with a diagnosis of SUD abuse or dependence (SUD Dx), **or** filled prescription for medications commonly used to treat addition	Source: HEDIS measure specifications (2018) QRS Measure Technical Specifications. Detoxification is not considered to be SUD treatment
ICD-9/10 codes for SUD Dx	See value set "Comprehensive SUD Dx"	
Medications		

Prescription	See "Med for OUD", "Med for AUD", "Med for Nicotine" and "Med for Cocaine" in the NDC directory	
Injectable or Service	Injectable naltrexone was identified based on a HCPCS code (J2315) in the medical claims. Buprenorphine service or implant can be identified with HCPCS codes J0570, J0571, J0572, J0573, J0574, J0575, and J0592. As methadone may only be used for MAT through in-office dispensing in licensed treatment centers, the methadone (OTP) service was also identified based on a HCPCS code (H0020 and S0109).	
Definition of Episode	For inpatient and residential treatment, an episode is defined as any sequential claims of the same modality with 0 or 1 days between the discharge date of the first claim and the admission date of the second claims. For outpatient and methadone claims, we determined that a new episode had been initiated whenever there was an interval of 45 or more days between successive visits. For codes, use values set: "Inpatient stay", "ED" with SUD Dx", "Detox" and "AOD procedures"	Shepard et al. (2002)
Definition of Relapse within an episode	For inpatient and residential treatment, an admission to the same level (meaning in ED, inpatient treatment with a primary diagnosis of a SUD or detoxification) of care within 2 to 30 days, is considered as 30-day relapse. For codes, use values set: "Inpatient stay", "ED" with SUD Dx", "Detox" and "AOD procedures"	Shepard et al. (2002), Clark 2011
	For outpatient or methadone services, an admission to inpatient or residential treatment as well to the same level of care (i.e., outpatient with a primary diagnosis of SUD or methadone services) within 2 to 30 days, it is considered as a relapse	Shepard et al. (2002), Clark 2011
Identification of methadone services	Filter "nonproprietary" column under the Med for OUD in the NDC directory. Select those that contain methadone	
**Outcome**	**Treatment Continuation**	**Notes**
**Sample inclusion criteria**		
Age	18-64	
Enrollment time	Enrolled at least one month in a given calendar year	
SUD condition	Had acute SUD treatment at any time in the enrolled year	
Notes		

Measure		
Numerator	Number of eligible populations who continued treatment for 3 months or more in the first six months of their eligible enrollment episode.	
Denominator	Number of adult enrollees who had SUD and treatment in the first six months of their eligible enrollment episode.	
Notes	Create binary indicator equals one if a member continued SUD treatment based on the codes provided below and 0 otherwise. Then calculate the mean or % of 1s per plan per year.	
**References**		
	Daley MC. Race, managed care, and the quality of substance abuse treatment. Adm Policy Ment Health. 2005;32(4):457-476	Source of the 7-30 day determination of rapid re-admission.
	Shepard DS et al., “Managed Care and the Quality of Substance Abuse Treatment,” Journal of Mental Health Policy and Economics. 2002; 5(4):163 –174.	Defines Episode and readmission
**Claims data code:**		
SUD Treatment (Tx)	Inpatient, intensive outpatient, partial hospitalization, outpatient, telehealth, detoxification, or ED visit with a diagnosis of SUD abuse or dependence (SUD Dx), **or** filled prescription for medications commonly used to treat addition	Source: HEDIS measure specifications (2018) QRS Measure Technical Specifications. Detoxification is not considered to be SUD treatment
ICD-9/10 codes for SUD Dx	See value set "Comprehensive SUD Dx"	
Medications		
Prescription	See "Med for OUD", "Med for AUD", "Med for Nicotine” and "Med for Cocaine” in the NDC directory	

Injectable or Service	Injectable naltrexone was identified based on a HCPCS code (J2315) in the medical claims. Buprenorphine service or implant can be identified with HCPCS codes J0570, J0571, J0572, J0573, J0574, J0575, and J0592. As methadone may only be used for MAT through in-office dispensing in licensed treatment centers, the methadone (OTP) service was also identified based on a HCPCS code (H0020 and S0109).	
Definition of Episode	For inpatient and residential treatment, an episode is defined as any sequential claims of the same modality with 0 or 1 days between the discharge date of the first claim and the admission date of the second claims. For outpatient and methadone claims, we determined that a new episode had been initiated whenever there was an interval of 45 or more days between successive visits. For codes, use values set: "Inpatient stay", "ED" with SUD Dx, "Detox" and "AOD procedures"	Shepard et al. (2002)
Treatment Continuity	For inpatient and residential treatment, an admission to a lower level (outpatient, methadone, or a lower level of residential) within 14 days of discharge from a first inpatient or residential episode, is considered as continuity of care.	Shepard et al. (2002)
	For outpatient or methadone treatment, admission to the same level (outpatient, methadone) within 14 days, is considered as continuity of care.	Shepard et al. (2002)
	If a client received outpatient or methadone services concurrently with a residential or inpatient treatment episode, continuity was achieved only if the outpatient or methadone visits extended beyond the discharge date for the residential or inpatient episode.	Shepard et al. (2002)
**Indicator**	**Social Connectedness**	**Notes**
**Sample inclusion criteria**		
Age	18-64	
Enrollment time	Enrolled at least one month in a given calendar year	
SUD condition	Had SUD in a given year	
**Measure**		
Numerator	Number of eligible population who received any peer-support service in the first six months of their eligible enrollment episode	

Denominator	Number of adult enrollees who had SUD in the first six months of their eligible enrollment episode.	
How to operationalize	Create binary indicator equals one if a member had received any peer-support service based on the codes provided below and 0 otherwise. Then calculate the mean or % of 1s per plan per year.	
**References**		
	Murrin S. Memorandum report: SAMHSA has improved outcome reporting for the substance abuse prevention and treatment block grant, oei-04-12-00160. 2015. Washington, DC: Substance Abuse and Mental Health Services Administration	Definition of indicator
	Chapman SA, Blash LK, Mayer K, Spetz J. Emerging Roles for Peer Providers in Mental Health and Substance Use Disorders. Am J Prev Med. 2018;54(6 Suppl 3):S267-S274.	Peer-support codes
**Claims data code:**		
Peer-support service code	H0038 (peer support – one-on-one)	Chapman et al. (2018)
	H0038 HQ (peer support – group)	
	H0038 GT (peer support – telephonic)	
	H2016 (comprehensive community support services – 3 or more hours in duration)	
	H2014 (skills training and development)	
	H2017 (peer support – telephonic)	
	H0025 (behavioral health prevention education service)	

eTable 2. SUD Population Identification

	Indicator	SUD population
Access		
1	Screening and 1st assessment visits - outpatient care	For this indicator we aimed to include all individuals that received an outpatient SUD services for the first six months of their eligible enrollment episode.
2	Presence of a substance use disorder diagnosis in claim (ASAM)	From all members from Medicaid, members who had at least one SUD Dx, or had at least one prescription for medications commonly used to treat addiction or were administered detoxification or rehabilitation procedures specifically for alcohol or drug disorder treatment in any outpatient, inpatient, or ED claims.
3	Any SUD service use for the 1st time	New episode of SUD during the Intake Period: Members who have an SUD event (Dx, prescription, or SUD CPT code) in the first six months of their eligible enrollment episode and are continuously enrolled in Medicaid for two months prior, and one month after (continuous enrollment time).
4	Tobacco Screening	From all Medicaid members, members who also received a tobacco screen in the first six months of their eligible enrollment episode.
Patterns of Care		
5	Follow-up after Withdrawal (Detoxification) Management	Had SUD withdrawal management episodes at any time in the enrolled year: (For 7 and 14 days)
6	SUD Treatment Initiation	People with SUD claims on prescription for a SUD medication, SUD diagnostic or SUD CPT claims including outpatient, inpatient,
7	SUD Treatment Engagement	People with a new episode of SUD during the Intake period: January 1 – November 15 of the measurement year. The Intake Period is used to capture new episodes including inpatient unit, outpatient, received SUD prescription or diagnostic.
8	Engagement: Alcohol Pharmacotherapy	People with a new episode of AUD during the Intake period: January 1 – November 15 of the measurement year. The Intake period is used to capture

		new episodes including inpatient unit, outpatient, received AUD prescription or diagnostic.
9	Engagement: Opioid Pharmacotherapy	Individuals who initiate Opioid specific pharmacotherapy with at least one prescription for an opioid treatment medication within 30 days following index visit with a diagnosis of opioid dependence abuse.
10	Smoking Cessation Engagement: Prescription/Consult; Treatment	Had a smoking related diagnosis at any time in the first six months of their eligible enrollment episode.
11	SUD Tx Engagement: Follow-up w/in 30 Days After ED Visit	Individuals with an ED visit (ED Value Set) with a principal diagnosis of AOD abuse or dependence (AOD Abuse and Dependence Value Set) on or between January 1 and December. (Some exclusions apply, see the table with algorithm construction components). For 7- and 30-days follow-up.
12	Identification of Comorbidity with Mental Illness	Individuals who had an SUD diagnosis/service at any time in the enrolled year.
13	Monthly expenditures on SUD Tx (Mean, including zeros)	Individuals treated for SUD in the current month. SUD treatment defined as either an ICD-9 or ICD-10 code and CPT for procedures: Inpatient, intensive outpatient, partial hospitalization, outpatient, telehealth, detoxification, or ED visit with a diagnosis of SUD abuse or dependence (SUD Dx), or filled prescription for medications commonly used to treat addiction
14	Percentage of non-zero expenditures on SUD Tx	Individuals treated for SUD in the current month. SUD treatment involved: Inpatient, intensive outpatient, partial hospitalization, outpatient, telehealth, detoxification, or ED visit with a diagnosis of SUD abuse or dependence (SUD Dx) or filled prescription for medications commonly used to treat addiction. Diagnosis of SUD (ICD-9, ICD-10) and SUD Prescription.
15	Receipt of Psychosocial interventions	From individuals that had an episode of SUD (treatment, diagnosis, or prescription) in a given year.
Outcomes		
16	Rapid Readmission to Inpatient SUD Care 2-30 days post-Discharge	Had acute SUD treatment at any time in the first six months of their eligible enrollment episode.
17	Relapse	Had SUD in the first six months of their eligible enrollment episode.
18	Treatment Continuation	Had acute SUD treatment at any time in the enrolled year. Number of eligible

		populations who continued treatment for 3 months or more in the first six months of their eligible enrollment episode: Inpatient, intensive outpatient, partial hospitalization, outpatient, telehealth, detoxification, or ED visit with a diagnosis of SUD abuse or dependence (SUD Dx) or filled prescription for medications commonly used to treat addition, SUD diagnosis or SUD prescription.
19	Social Connectedness	Had SUD in a given year (Dx, Treatment, prescription)

eTable 3. Plan Performance on Quality of Substance Use Disorder Treatment (Full Sample)				
**Values Shown as Percentages**				
**Indicator**	**Mean (range) (2009-2011)**	**Mean (range) (2012-2014)**	**Mean (range) (2015-2017)**	**Mean (range) (Aggregate)**
Access				
Screening and 1st assessment visits – outpatient care	5.3 (5.2, 5.3)	7.6 (7.5, 7.6)	10.0 (9.9, 10.1)	7.2 (7.2, 7.3)
Presence of an SUD diagnosis in claim	7.6 (7.6, 7.7)	8.6 (8.5, 8.6)	9.5 (9.5, 9.6)	8.5 (8.4, 8.5)
Any SUD service use for the 1st time	7.5 (7.5, 7.6)	8.5 (8.4, 8.5)	9.4 (9.4, 9.5)	8.4 (8.3, 8.4)
Tobacco Screening	1.4 (1.4, 1.4)	2.7 (2.7, 2.8)	3.3 (3.3, 3.3)	2.4 (2.4, 2.4)
Patterns of Care				
Follow up after Withdrawal (Detoxification) Management (14 days)	12.4 (11.9, 12.8)	15.4 (14.9, 15.8)	17.5 (16.9, 18.1)	14.9 (14.7, 15.2)
SUD Tx Initiation	44.6 (43.0, 46.3)	41.2 (39.7, 42.7)	37.5 (36.2, 38.8)	40.6 (39.8, 41.5)
SUD Tx Engagement	0.7 (0.5, 1.0)	2.4 (1.9, 2.9)	6.7 (6.0, 7.3)	3.7 (3.4, 4.0)
Engagement: Alcohol Pharmacotherapy	0.7 (0.3, 1.0)	0.9 (0.5, 1.3)	1.6 (1.2, 1.9)	1.2 (1.0, 1.4)
Engagement: Opioid Pharmacotherapy	24.8 (23.1, 26.5)	33.1 (31.6, 34.6)	24.0 (22.7, 25.2)	27.4 (26.6, 28.3)
Smoking Cessation Engagement	0.6 (0.5, 0.7)	0.8 (0.7, 0.8)	4.3 (4.0, 4.5)	1.8 (1.8, 1.9)
SUD Tx Engagement: Follow-up w/in 30 days after ED visit	22.8 (20.8, 24.8)	22.5 (20.9, 24.2)	20.1 (18.9, 21.3)	21.4 (20.5, 22.3)
Identification of comorbidity with mental illness	14.7 (14.4, 14.9)	15.3 (15.1, 15.5)	16.0 (15.8, 16.3)	15.3 (15.2, 15.4)
Monthly Expenditures on SUD Tx (Mean, including zeros)	$68.9 ($67.5, $70.3)	$116.4 ($114.4, $118.4)	$212.7 ($208.9, $216.5)	$123.1 ($121.7, $124.4)

Percentage of non-zero expenditures on SUD Tx	5.1 (5.1, 5.2)	6.9 (6.9, 7.0)	7.3 (7.3, 7.4)	6.4 (6.3, 6.4)
Receipt of Psychosocial interventions	43.7 (43.4, 44.0)	32.4 (32.2, 32.7)	30.7 (30.4, 31.0)	35.7 (35.5, 35.9)
Outcomes				
Rapid Readmission to Inpatient SUD Care 2-30 days post-discharge	17.2 (16.7, 17.8)	20.5 (20.0, 21.0)	19.6 (19.0, 20.5)	19.2 (18.9, 19.5)
Relapse (2-30 days)	9.3 (9.1, 9.5)	9.7 (9.5, 9.8)	20.5 (20.3, 20.8)	12.6 (12.5, 12.8)
Treatment Continuation	2.9 (2.8, 3.0)	2.7 (2.6, 2.7)	9.2 (9.1, 9.4)	4.6 (4.5, 4.7)
Social Connectedness (peer services)	0.7 (0.6, 0.7)	0.6 (0.6, 0.7)	0.3 (0.3, 0.4)	0.6 (0.5, 0.6)
Abbreviation: SUD, Substance Use Disorder; Tx, Treatment.				

eTable 4. Regression – Plan Effects on Quality of SUD Treatment (Full Sample)

Values Shown as Percentages

**Indicator**	**Plan 1**	**Plan 2**	**Plan 3**	**Plan 4**	**Plan 5**	**Plan 6**	**Plan 7**	**Plan 8**	**Plan 9**
Access									
Screening and 1st SUD assessment visits - outpatient care	-0.1	0.0	0.2[c]	-0.6[c]	-0.5[c]	0.0	-0.7[c]	0.7[c]	0.5[c]
Presence of SUD diagnosis in claim	-0.9[c]	2.1[c]	-0.8[c]	-2.3[c]	2.6[c]	0.1[b]	-1.9[c]	-0.5[c]	-2.8[c]
Any SUD service use for the 1st time	-0.8[c]	2.0[c]	-0.7[c]	-2.3[c]	2.5[c]	0.1[b]	-1.8[c]	-0.5[c]	-2.8[c]
Tobacco Screening	-0.2[c]	0.7[c]	0.2[c]	-0.5[c]	0.7[c]	-0.7[c]	-0.8[c]	-0.6[c]	0.0
Patterns of Care									
Follow up after Withdrawal (Detox) Management - 14 days	-3.1[c]	-1.5[c]	0.3	-1.4[b]	0.2	8.2[c]	-3.2[c]	-0.6	-2.9[b]
SUD Tx Initiation	2.9	-0.2	-0.3	7.9[c]	-5.1[b]	7.9[c]	1.0	-9.8[c]	-4.6[a]
SUD Tx Engagement	-0.1	-0.3	0.8[a]	0.6	-0.6	0.5	-1.1	-0.7	0.5
Engagement: Alcohol Pharmacotherapy	0.4	0.3	-0.4	-0.2	0.0	0.0	0.4	0.1	-1.3
Engagement: Opioid Pharmacotherapy	-2.4	-1.8[b]	2.4[a]	2.0	-2.8	4.1[b]	1.3	-0.3	3.4
Smoking Cessation Engagement	-0.7[c]	0.8[c]	-0.8[c]	-0.7[c]	-0.3	0.1	-0.2	0.2	-0.5[a]
SUD Tx Engagement: Follow-up w/in 30 days of ED visit	-3.4	-0.4	0.9	0.5	1.5	4.1[b]	-11.4[b]	-0.5	-7.1[b]
Identification of comorbid mental illness	-1.2[c]	-0.2	1.4[c]	-0.5[a]	-0.2	2.8[c]	0.1	-2.3[c]	-2.8[c]
Monthly expenditures on SUD Tx (Mean, including zeros)	-$31.9[c]	$0.7	-$8.1[c]	-$36.9[c]	$5.0	$120.8[c]	-$44.4[c]	-$11.0[c]	$27.4[c]
Receipt of Psychosocial interventions	0.1	1.1[c]	-1.1[c]	-2.5[c]	0.3	3.2[c]	-2.7[c]	-0.3	-9.2[c]
Outcomes									

Rapid Readmission to Inpatient SUD Care 2-30 days post-discharge	-0.2	-4.1[c]	1.3[c]	3.0[c]	2.9[c]	7.5[c]	-3.1[c]	-2.7[b]	0.6[c]
Relapse (2-30 days)	-0.7[b]	0.1	1.1[c]	-0.3	-1.9[c]	5.9[c]	-3.2[c]	-4.5[c]	-0.1
Treatment Continuation	-0.3[a]	0.0	1.1[c]	-0.4[b]	-0.7[c]	2.1[c]	-1.3[c]	-2.1[c]	-0.6[a]
Social Connectedness (peer services)	0.1[a]	0.1[c]	0.0	-0.2[c]	0.0	-0.2[c]	0.2[a]	-0.1	-0.1

Abbreviation: SUD, Substance Use Disorder; Tx, Treatment.

[a] $p < .05$

[b] $p < .01$

[c] $p < .001$

Positive numbers reflect better performance than the average performance across all 9 plans, and negative numbers indicate worse performance.

eTable5. STROBE Statement - Checklist of items that should be included in reports of *cross-sectional studies*

	Item No	Recommendation	Page No
**Title and abstract**	1	(*a*) Indicate the study's design with a commonly used term in the title or the abstract	1
		(*b*) Provide in the abstract an informative and balanced summary of what was done and what was found	1
**Introduction**			
Background/rationale	2	Explain the scientific background and rationale for the investigation being reported	2
Objectives	3	State specific objectives, including any prespecified hypotheses	2
**Methods**			
Study design	4	Present key elements of study design early in the paper	2-3
Setting	5	Describe the setting, locations, and relevant dates, including periods of recruitment, exposure, follow-up, and data collection	3
Participants	6	(*a*) Give the eligibility criteria, and the sources and methods of selection of participants	3
Variables	7	Clearly define all outcomes, exposures, predictors, potential confounders, and effect modifiers. Give diagnostic criteria, if applicable	3
Data sources/ measurement	8*	For each variable of interest, give sources of data and details of methods of assessment (measurement). Describe comparability of assessment methods if there is more than one group	*3-4*
Bias	9	Describe any efforts to address potential sources of bias	3
Study size	10	Explain how the study size was arrived at	3
Quantitative variables	11	Explain how quantitative variables were handled in the analyses. If applicable, describe which groupings were chosen and why	3-5
Statistical methods	12	(*a*) Describe all statistical methods, including those used to control for confounding	3-5
		(*b*) Describe any methods used to examine subgroups and interactions	4-5
		(*c*) Explain how missing data were addressed	5
		(*d*) If applicable, describe analytical methods taking account of sampling strategy	5 (and

			Supplement)
		(*e*) Describe any sensitivity analyses	4-5 (and Supplement)
**Results**			
Participants	13*	(a) Report numbers of individuals at each stage of study—eg numbers potentially eligible, examined for eligibility, confirmed eligible, included in the study, completing follow-up, and analyzed	3
		(b) Give reasons for non-participation at each stage	3
		(c) Consider use of a flow diagram	n/a
Descriptive data	14*	(a) Give characteristics of study participants (eg demographic, clinical, social) and information on exposures and potential confounders	5
		(b) Indicate number of participants with missing data for each variable of interest	n/a
Outcome data	15*	Report numbers of outcome events or summary measures	Table 1
Main results	16	(*a*) Give unadjusted estimates and, if applicable, confounder-adjusted estimates and their precision (eg, 95% confidence interval). Make clear which confounders were adjusted for and why they were included	Table 1,2,3; page 6
		(*b*) Report category boundaries when continuous variables were categorized	
		(*c*) If relevant, consider translating estimates of relative risk into absolute risk for a meaningful time period	
Other analyses	17	Report other analyses done—eg analyses of subgroups and interactions, and sensitivity analyses	5-6
**Discussion**			
Key results	18	Summarize key results with reference to study objectives	7-8
Limitations	19	Discuss limitations of the study, taking into account sources of potential bias or imprecision. Discuss both direction and magnitude of any potential bias	9
Interpretation	20	Give a cautious overall interpretation of results considering objectives, limitations, multiplicity of analyses, results from similar studies, and other relevant evidence	7-9
Generalizability	21	Discuss the generalizability (external validity) of the study results	10

**Other information**			
Funding	22	Give the source of funding and the role of the funders for the present study and, if applicable, for the original study on which the present article is based	10

*Give information separately for exposed and unexposed groups.
